# Direct cortical thickness estimation using deep learning‐based anatomy segmentation and cortex parcellation

**DOI:** 10.1002/hbm.25159

**Published:** 2020-08-12

**Authors:** Michael Rebsamen, Christian Rummel, Mauricio Reyes, Roland Wiest, Richard McKinley

**Affiliations:** ^1^ Support Center for Advanced Neuroimaging (SCAN), University Institute of Diagnostic and Interventional Neuroradiology University of Bern, Inselspital, Bern University Hospital Bern Switzerland; ^2^ Graduate School for Cellular and Biomedical Sciences University of Bern Bern Switzerland; ^3^ Insel Data Science Center, Inselspital Bern University Hospital Bern Switzerland; ^4^ ARTORG Center for Biomedical Research University of Bern Bern Switzerland

**Keywords:** brain morphometry, cortical thickness, deep learning, diffeomorphic registration, gray matter atrophy, MRI, neuroanatomy segmentation

## Abstract

Accurate and reliable measures of cortical thickness from magnetic resonance imaging are an important biomarker to study neurodegenerative and neurological disorders. Diffeomorphic registration‐based cortical thickness (DiReCT) is a known technique to derive such measures from non‐surface‐based volumetric tissue maps. ANTs provides an open‐source method for estimating cortical thickness, derived by applying DiReCT to an atlas‐based segmentation. In this paper, we propose DL+DiReCT, a method using high‐quality deep learning‐based neuroanatomy segmentations followed by DiReCT, yielding accurate and reliable cortical thickness measures in a short time. We evaluate the methods on two independent datasets and compare the results against surface‐based measures from FreeSurfer. Good correlation of DL+DiReCT with FreeSurfer was observed (*r* = .887) for global mean cortical thickness compared to ANTs versus FreeSurfer (*r* = .608). Experiments suggest that both DiReCT‐based methods had higher sensitivity to changes in cortical thickness than Freesurfer. However, while ANTs showed low scan‐rescan robustness, DL+DiReCT showed similar robustness to Freesurfer. Effect‐sizes for group‐wise differences of healthy controls compared to individuals with dementia were highest with the deep learning‐based segmentation. DL+DiReCT is a promising combination of a deep learning‐based method with a traditional registration technique to detect subtle changes in cortical thickness.

## Graphical abstract

Accurate and reliable measures of cortical thickness from magnetic resonance imaging (MRI) are essential biomarkers to study neurodegenerative and neurological disorders. We propose DL+DiReCT, a method to measure cortical thickness from structural MRI by combining a deep learning‐based neuroanatomy segmentation and cortex parcellation with a traditional diffeomorphic registration technique. Extended validation experiments on two large scale datasets suggest the method is sensitive to detect subtle changes in cortical thickness and comparably robust to surface‐based measurement as used by FreeSurfer.

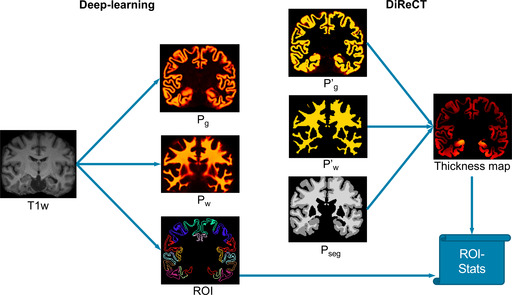

## 1 INTRODUCTION

The human cerebral cortex, a thin ribbon of gray matter constituting the outer layer of the cerebrum, is on average about 2.5 mm thick (Fischl & Dale, 2000). Cortical thickness decreases with normal aging (Salat et al., 2004), a process that is known to be accelerated in neurodegenerative diseases including dementia (Young et al., 2020). The pattern of atrophy progression may enable to differentiate the underlying form of dementia, but also to characterize mild cognitive impairment (Karas et al., 2004). In the case of Alzheimer's disease (AD), the onset is usually located in the transentorhinal cortex and extending into the temporal lobe (Braak & Braak, 1991; Kulason et al., 2019) before spreading to other regions in the brain in a well‐defined sequence in later stages of the disease (Thompson et al., 2003). Numerous studies have demonstrated that cortical thickness can serve as a surrogate marker for the underlying pathological changes (Frisoni, Fox, Jack, Scheltens, & Thompson, 2010; Lerch et al., 2005; Singh et al., 2006; Whitwell et al., 2008). Quantitative morphometry and its regional patterns of atrophy are therefore considered a potential biomarker of clinical interest (Dickerson et al., 2009; Young et al., 2020).

However, measuring the cortical thickness from magnetic resonance imaging (MRI) with sub‐voxel accuracy is a difficult task. Modeling the cortical band as a surface mesh to calculate the thicknesses has been shown (Fischl & Dale, 2000) to be a capable technique and is available in the popular FreeSurfer (Fischl, 2012) software. Surface‐based methods are also employed in tools like BrainSuite (Shattuck & Leahy, 2002), BrainVISA (Mangin, Frouin, Bloch, Régis, & López‐Krahe, 1995), or CIVET (Lerch & Evans, 2005; MacDonald, Kabani, Avis, & Evans, 2000). Alternative methods like using Laplace's Equations (Jones, Buchbinder, & Aharon, 2000) or registration‐based solutions (Das, Avants, Grossman, & Gee, 2009) have been proposed. The accuracy of these methods has been evaluated and compared to FreeSurfer by others (Clarkson et al., 2011; Tustison et al., 2014).

Registration‐based solutions rely on good tissue segmentation of white‐matter (WM), gray‐matter (GM), and cortical (sulcal) cerebrospinal fluid (CSF). Under the assumption that the interfaces of WM/GM and GM/CSF share a common topology, the WM/GM boundary is deformed toward the GM/CSF boundary using a diffeomorphic registration and a thickness map is derived from the distance between corresponding points (Das et al., 2009). An open‐source implementation of this *diffeomorphic registration based cortical thickness* (DiReCT) algorithm is available in ANTs (Avants et al., 2014) as part of the ANTs cortical thickness pipeline (Tustison et al., 2013) which applies DiReCT to segmentations derived from Atropos (Avants, Tustison, Wu, Cook, & Gee, 2011), an atlas‐based segmentation method.

Deep‐learning (DL) (LeCun, Bengio, & Hinton, 2015) is a promising technique for medical image analysis (Litjens et al., 2017), with image segmentation currently being the most used application of DL in neuroimage analysis (Yao, Cheng, Pan, & Kitamura, 2020). However, the adoption of deep neural networks for advanced tasks like extraction of biomarkers or direct prediction of diagnosis is challenged by the lack of interpretability, especially for clinical applications where trust in the model and traceability of the results is required (Ching et al., 2018).

**FIGURE 1:**
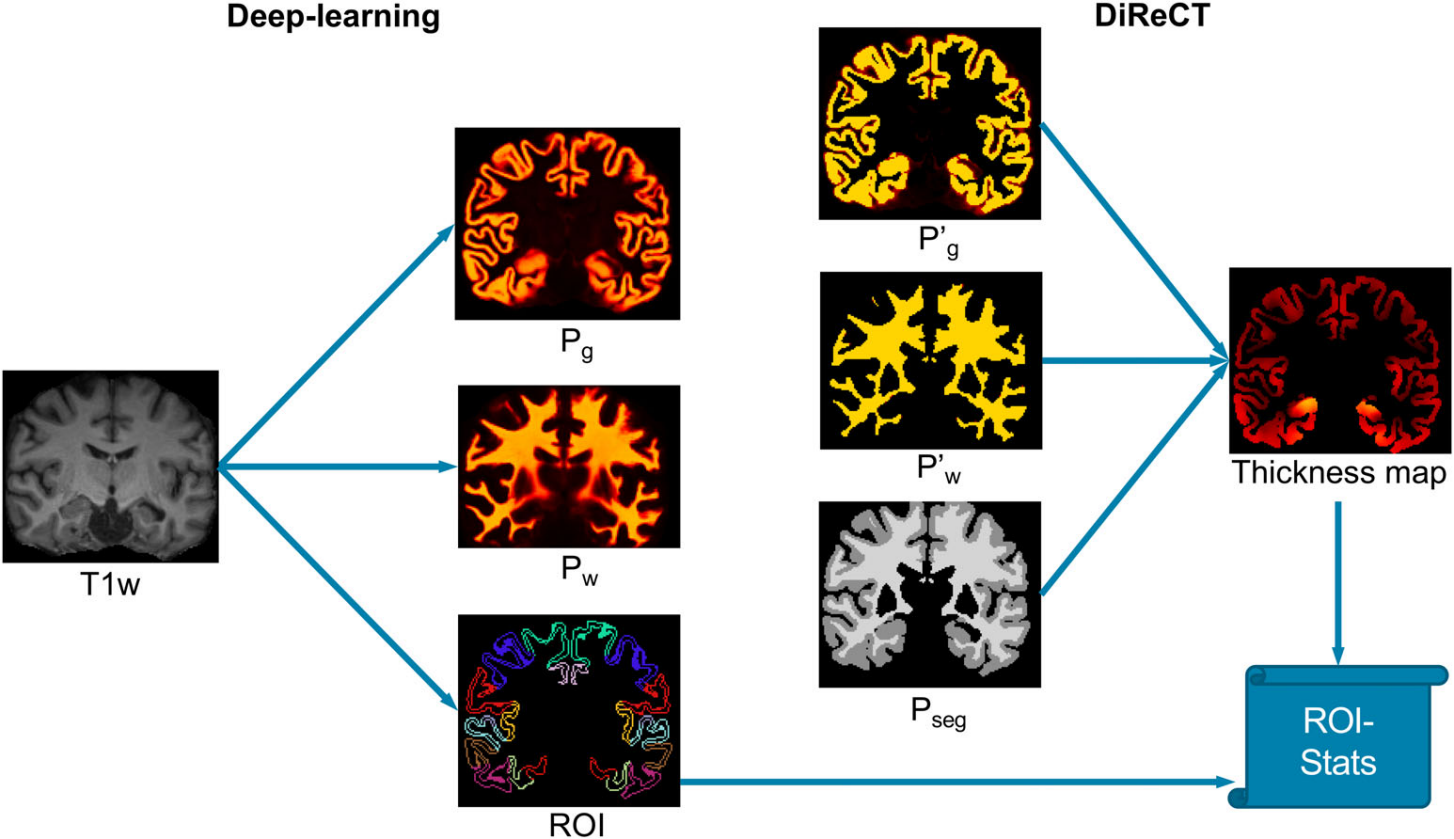
DL+DiReCT: Deep learning‐based neuroanatomy segmentation followed by a diffeomorphic registration to estimate cortical thickness from MRI

For neuroanatomy segmentation, numerous recent publications have shown the superiority of DL over traditional methods (Dalca, Balakrishnan, Guttag, & Sabuncu, 2019; Roy et al., 2019; Wachinger, Reuter, & Klein, 2018). We hypothesize that registration‐based thickness measures benefit from more accurate and reliable segmentations than usually available from atlas‐based methods. Therefore, we propose DL+DiReCT: A Deep learning‐based anatomy segmentation including parcellation of the cortex followed by DiReCT to derive cortical thickness measures directly from T1‐weighted (T1w) MRI as outlined in Figure 1. We demonstrate its reliability with extended validation experiments on two datasets and show its potential to detect regional patterns of atrophy in dementia patients.

## 2 MATERIALS AND METHODS

### 2.1 Deep learning‐based anatomy segmentation and cortex parcellation

For DL+DiReCT we used DeepSCAN, an in‐house developed Deep learning‐based model for neuroanatomy segmentation (McKinley et al., 2019; McKinley et al., 2019). The network architecture is, in brief, based on densely connected blocks of dilated convolutions (Huang, Liu, Van Der Maaten, & Weinberger, 2017) in a U‐net (Ronneberger, Fischer, & Brox, 2015) like structure. The model was trained with a total of 840 T1w MRI by combining publicly available and internal data. From public datasets, we used data from 160 nine to ten year old children from ABCD (Casey et al., 2018), 160 healthy adults from IXI (brain-development.org/ixi-dataset), and 160 elderly people from ADNI (Jack Jr et al., 2008). Internal data from our institution (Inselspital) from previous studies comprised 160 healthy controls, 128 patients with multiple sclerosis, 48 patients with epilepsy, and 24 cases with Parkinson's disease. The input data was minimally preprocessed (see Section 2.2.1). For the supervised training, the following 96 weak labels from FreeSurfer 6.0 were used: left/right cerebral white matter, cortical gray matter including its Desikan‐Killiany parcellations (Desikan et al., 2006), lateral ventricle, cerebellum (WM + GM), accumbens area, amygdala, caudate, hippocampus, pallidum, putamen, thalamus, ventral DC, and the central structures 3^rd^ ventricle, 4^th^ ventricle, brainstem, and corpus callosum. As we use a multi‐label classification scheme accounting for class imbalances (Cui, Jia, Lin, Song, & Belongie, 2019), a voxel may be assigned more than one label, which allows robust identification of the cortical gray matter and an independent assignment of parcellation labels. The model was trained with focal loss (Lin, Goyal, Girshick, He, & Dollár, 2017) and a cosine annealing learning rate schedule for 100 epochs and a batch size of two.

### 2.2 Cortical thickness estimation

#### 2.2.1 FreeSurfer

Results for FreeSurfer were generated using the *recon‐all* pipeline of FreeSurfer 6.0 (Fischl, 2012) running on Linux on a single CPU. No manual corrections were made. Regional mean cortical thickness was extracted from the surface statistics (*lh.aparc.stats*, *rh.aparc.stats*).

Using the output from FreeSurfer, minimal preprocessing of the original T1w images was performed: resampling into FreeSurfer's space (*mri_vol2vol*) followed by an application of the brain mask from FreeSurfer. These 1 mm iso‐voxel images with skull‐stripped brains serve as input for the two methods below.

#### 2.2.2 ANTs

The results for ANTs were generated with the default cortical thickness pipeline (*antsCorticalThickness.sh*) (Tustison et al., 2013) running on Linux restricted to a single CPU. The publicly available *OASIS‐30_Atropos_template* (Klein, 2016; Klein & Tourville, 2012) was used as population level template for Atropos (Avants et al., 2011). The resulting output is a voxel‐wise volumetric thickness map.

#### 2.2.3 DL+DiReCT

Preliminary results by directly using the probability maps from the DL model as input for DiReCT suggested that preprocessing is required such that the input is more alike a hard segmentation. In particular, we actually used a binary image for the WM as suggested by Clarkson et al. (2011), which is reasonable knowing that the WM is the moving image in the registration that does not change topology (Das et al., 2009).

Where *P_*w*_* is the *sigmoid* of the *logit* output for the classification of WM labels, and equivalently *P_*g*_* for GM by taking the maximum *logit* of the cortical GM, Amygdalae, and Hippocampi labels, we calculated the input for DiReCT for every voxel *x* in the image volume as follows:

(1) $$P_{\mathrm{seg}}\left(x\right)=\left\{\begin{array}{cc} \text{argmax}\left(P_{g}\left(x\right),P_{w}\left(x\right)\right)+2, & \text{if}\;\left(P_{g}\left(x\right)+P_{w}\left(x\right)\right)>0.7\\ 2, & \text{if}\;P_{g}\left(x\right)>0.5\\ 3, & \text{if}\;P_{w}\left(x\right)>0.5\\ 0, & \text{otherwise} \end{array}\right)$$

(2) Pw'x=1,ifPwx>PgxandPsegx>00,otherwise

(3) Pg'x=1,ifPgx>0.5Pgx,ifPgx>Pwx0,otherwise

In the hard segmentation *P_*seg*_* (Equation (1)), 2 corresponds to GM and 3 to WM, and *argmax* returns the position of the largest element starting at index 0. The probability maps $P_{w^{\prime }}$ (Equation (2)) for WM and $P_{g^{\prime }}$ (Equation (3)) for GM were constructed such that there is a well‐defined WM/GM boundary. These preprocessing steps were determined empirically on an independent internal validation set. From these volumes, the thickness map of DL+DiReCT was calculated by a diffeomorphic registration using DiReCT with convergence settings identical to ANTs.

#### 2.2.4 Parcellation‐wise average cortical thickness

From the volumetric voxel‐wise thickness map of ANTs and DL+DiReCT, we calculated average cortical thickness statistics for regions of interest (ROI) according to the Desikan‐Killiany (DK) atlas (Desikan et al., 2006), providing 34 ROIs per hemisphere. For ANTs, we used the parcellation labels from FreeSurfer (*aparc+aseg*) and for DL+DiReCT the labels from the DL model. For DL+DiReCT we additionally calculated complementary results by also using the parcellation labels from FreeSurfer instead of the DL model. All voxels constituting the inner boundary of the gray‐matter segmentation were identified and assigned the label of the closest parcellation. Voxels further away than an Euclidean distance of $\sqrt{3.0}$ voxel dimensions were masked in order to exclude deep gray‐matter structures. Within this defined region of interest, the average over all nonzero voxel from the thickness map was calculated.

### 2.3 Data for evaluation

For evaluation, we used T1‐weighted (T1w) MRI from two publicly available datasets: OASIS‐3 (LaMontagne et al., 2018) and SIMON (Duchesne et al., 2019), yielding a total of 2,736 images. OASIS‐3 contains cross‐sectional and longitudinal samples from cognitively normal adults, as well as participants at various stages of dementia, as assessed by the Clinical Dementia Rating (CDR) (Morris, 1991). Data from SIMON stem from a single healthy male volunteer known as “the traveling human phantom,” providing repeated measures from different sites over a time span of 16 years. Demographic information is listed in Table 1. The images from OASIS‐3 were all acquired on three different models of Siemens scanners (1.5 T MAGNETOM Sonata, and 3T Biograph mMR and MAGNETOM Trio), whereas SIMON contains data from various models of Siemens, Philips, and GE. No data from the OASIS‐3 or SIMON datasets were used to train the brain anatomy segmentation model described above.

**TABLE 1:** Demographic information for the two datasets used for the evaluation

	# Subjects	Mean age (range)	# T1w	# per CDR				
				0	0.5	1	2	3
OASIS‐3	1,038	70.7 (42.7–97.0)	2,643	2014	420	159	40	10
SIMON	1	43.5 (29.7–46.4)	93	—	—	—	—	—

Abbreviation: CDR, clinical dementia rating.

### 2.4 Evaluation

We processed the MR images (2,643 from OASIS‐3 and 93 from SIMON) with all three methods, yielding 70 cortical thickness measures per image (34 ROI‐averages and mean thickness for left and right hemisphere). As a primary outcome measure of interest, we use the average mean thickness of the left and right hemisphere, which we refer to as global mean thickness in the manuscript. We considered subjects with CDR = 0 as healthy controls (HC), CDR = 0.5 as questionable, and CDR >  = 1 as confirmed dementia (Manning & Ducharme, 2010).

For assessing robustness, we used re‐scans where two or more images of an individual were acquired during the same session. Under the assumption that consecutive measures should ideally produce the same result and reflect reproducibility (Jovicich et al., 2013), we calculate for each measure *m* the average *absolute changes relative to the mean (%)*:

(4) $${ɛ}_{\mu }=\frac{100}{N}\sum_{i=1}^{N}\left(\frac{1}{n\left(i\right)}\sum_{t=1}^{n\left(i\right)}\frac{|m_{\left(i,t\right)}-\mu_{\left(i\right)}|}{\mu_{\left(i\right)}}\right)$$

where *N* is the number of sessions with re‐scans, *n*(*i*) the number of re‐scans in the session *i* for a subject, *m*_(*i*, *t*)_ the measurement at timepoint *t*, and $\mu_{\left(i\right)}=\frac{1}{n\left(i\right)}\sum_{t=1}^{n\left(i\right)}m_{\left(i,t\right)}$ the within‐session mean.

To regress out the effects of brain size, age, sex, and scanner on cortical thickness, we fit a linear model (lm) to the thickness of the healthy controls with the normalized (zero‐mean, unit SD) co‐variates *estimated total intracranial volume* (eTIV; Buckner et al., 2004; from FreeSurfer) and age, and categorical variables sex and scanner model. In agreement with Im et al. (2008) the co‐variate sex was not significantly related to thickness and was subsequently removed. Likewise, the scanner had no significant effect after accounting for multiple comparisons (Mundfrom, Perrett, Schaffer, Piccone, & Roozeboom, 2006), resulting in a lm(thick ∼ eTIV + age) that was then applied to *all* samples. On these thickness measures corrected for brain size and age, we calculated the effect size using Cohen's *d* (Torchiano, 2019) to quantify group‐wise differences between healthy controls (CDR = 0) and subjects with dementia (CDR >  = 1).

Additionally, we quantified longitudinal annual cortical GM atrophy rates separately for the three OASIS‐3 sub‐cohorts (HC, CDR = 0.5, and dementia) by using subjects who had more than one scan at least 1 year apart and who did not change sub‐cohort (e.g., from HC to dementia) in that interval. Atrophy rates between methods were compared with a paired *t*‐test and a significance level *α* = .05. Statistical analyses were performed using *R* with the *stats* package version 3.6.2 (R Core Team, 2019).

## 3 RESULTS

The deep learning‐based anatomy segmentation, when compared to FreeSurfer, reached median Dice coefficients above .97 for WM and above .95 for cortical GM on both datasets. Detailed performance for the relevant structures is reported in Supplementary Table S1. The average runtimes per image for the three methods were 9.34*h* ± 2.68*h* for FreeSurfer, 12.68*h* ± 0.90*h* for ANTs, and 1.18*h* ± 0.17*h* for DL+DiReCT.

**FIGURE 2:**
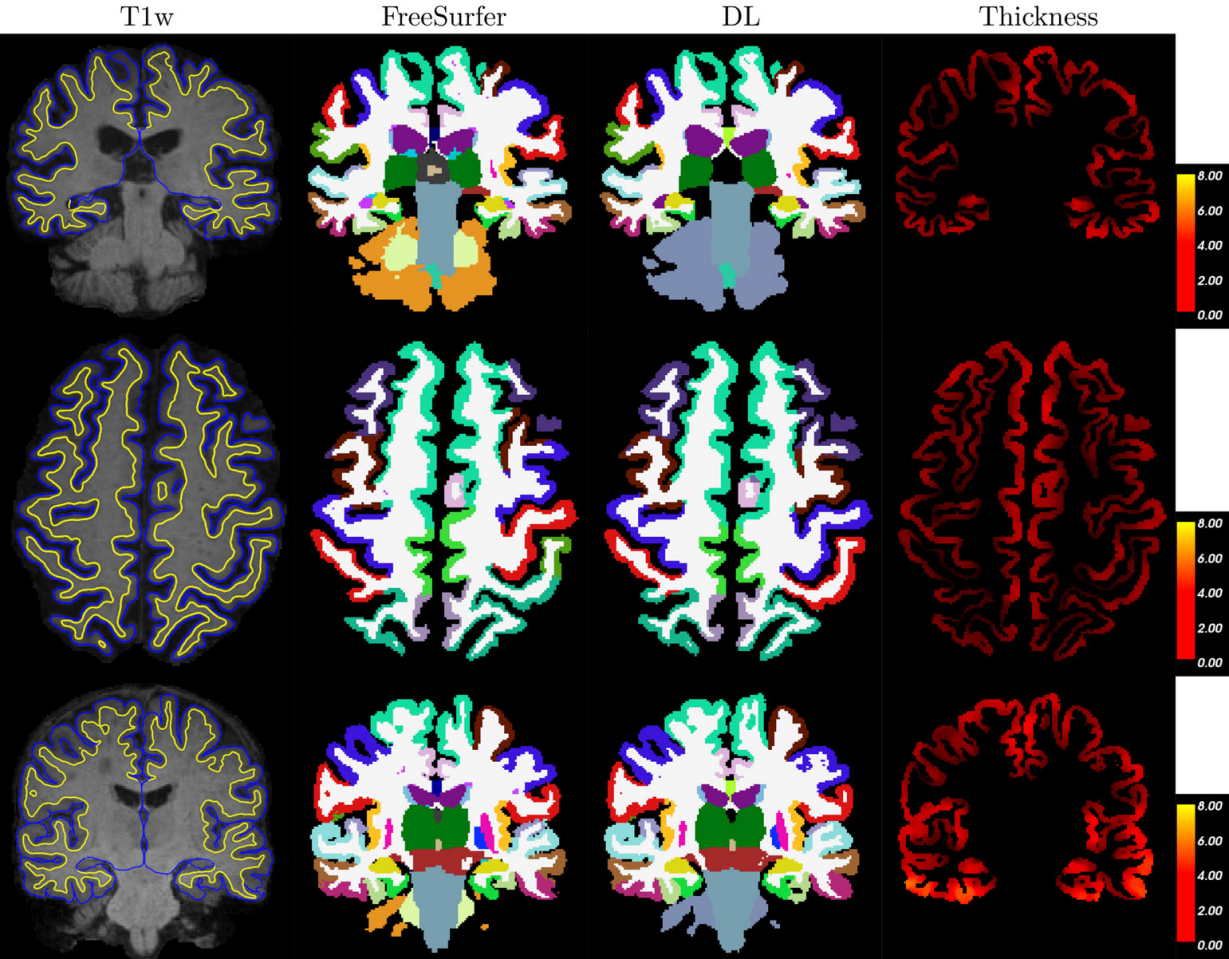
Three samples (one per row) from the OASIS‐3 dataset. Columns show T1‐weighted MRI with pial (blue) and GM/WM (yellow) surface from FreeSurfer overlayed, segmentations from FreeSurfer and deep learning (DL), and thickness map from DL+DiReCT. Slices are in radiological view (i.e., right hemisphere is on the left side of the image)

Three selected qualitative examples are shown in Figure 2. These cases were chosen from the OASIS‐3 dataset as follows: Best agreement between regional thickness measures between FreeSurfer and DL+DiReCT (highest Pearson correlation across all regions), largest absolute difference of the thickness measure in the left postcentral gyrus (FreeSurfer = 2.4 mm, DL+DiReCT = 1.5 mm), and largest difference in the left inferior temporal gyrus (FreeSurfer = 2.8 mm, DL+DiReCT = 3.9 mm). The large deviation for the thickness of the postcentral gyrus (cf., second row) was caused by a mislabeling where FreeSurfer erroneously identified the precentral gyrus (blue) as postcentral gyrus (red) in the left hemisphere.

### 3.1 Correlation with FreeSurfer

**FIGURE 3:**
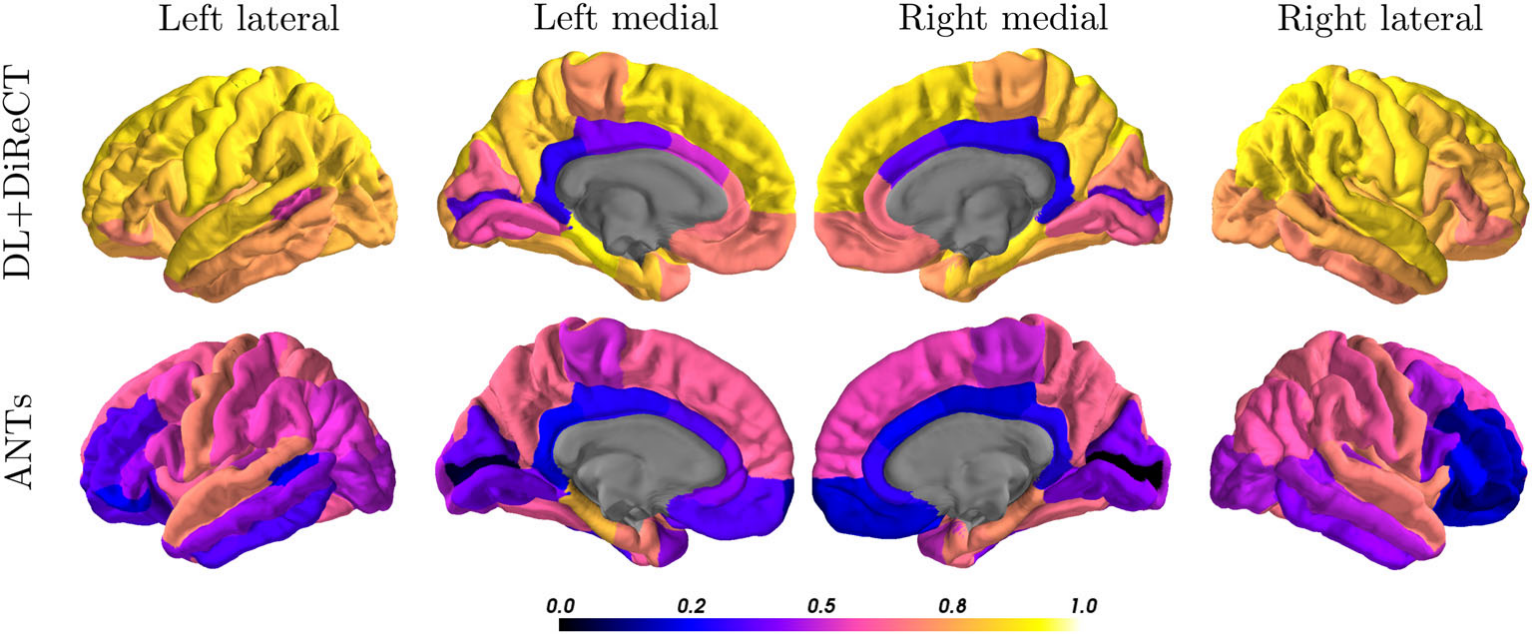
Color‐coded Pearson correlation coefficients (*r*) of the ROI‐wise average cortical thicknesses compared to FreeSurfer evaluated on the OASIS‐3 samples

On the OASIS‐3 dataset (*n* = 2,643 images), the global mean thickness of DL+DiReCT was Pearson correlated with FreeSurfer with *r* = .887 while the results of the same test for ANTs were *r* = .608. A visualization of the region‐wise correlation coefficients can be seen in Figure 3. For DL+DiReCT, the unweighted average over all ROIs was *r* = .716 and was highest in the parietal lobe (mean *r* = .836) followed by frontal (*r* = .763), temporal (*r* = .763), and occipital lobe (*r* = .599), and was lowest in the cingulate cortex (*r* = .440). Accordingly, the results for ANTs was *r* = .452 for the ROI‐average and for the lobes: parietal (*r* = .599), frontal (*r* = .391), temporal (*r* = .545), occipital (*r* = .329), and cingulate cortex (*r* = .297). For comparison, the ROI‐average for DL+DiReCT when using the FreeSurfer parcellation was *r* = .734. Remaining results for DL+DiReCT relate to the deep learning‐based parcellation unless noted otherwise.

**FIGURE 4:**
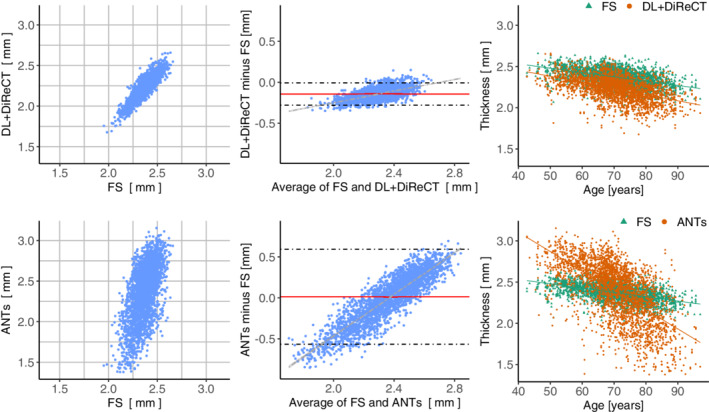
Comparison of the global mean thickness estimations against FreeSurfer (FS) for DL+DiReCT (first row) and ANTs (second row) for the samples in the OASIS‐3 dataset. Left: correlation plot. Middle: Bland–Altman plot, dashed horizontal line indicating ±1.96*σ*. Right: Thicknesses plotted against age

As can be seen in the Bland–Altman plots in Figure 4, both DiReCT‐based methods underestimate smaller thicknesses and overestimate the larger in comparison to FreeSurfer. This also results in larger cross‐sectional annual age‐related GM atrophy rates for DL+DiReCT (−0.007 mm/year) and ANTs (−0.023 mm/year) compared to FreeSurfer (−0.005 mm/year). Additional plots of thickness measures for all regions can be found in Section S5.

### 3.2 Robustness

**FIGURE 5:**
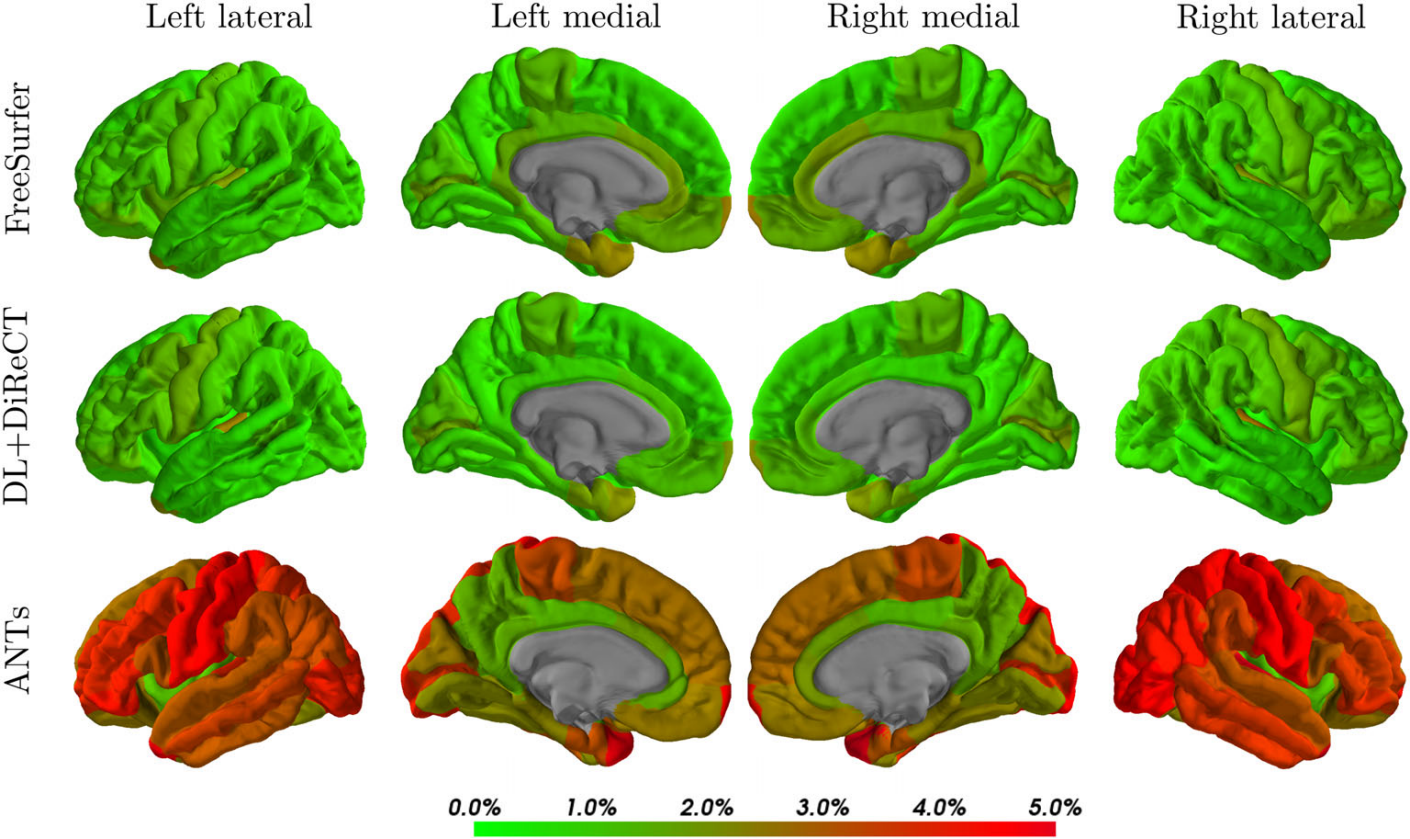
Color‐coded reproducibility errors of the ROI‐wise average cortical thicknesses evaluated on the OASIS‐3 samples

The mean reproducibility errors are listed in Table 2, for the global mean thickness measure and as an average over all 68 ROIs. On both datasets, OASIS‐3 (*n* = 761 sessions) and SIMON (*n* = 14), we observed similar errors for FreeSurfer and DL+DiReCT and significantly higher error for ANTs as can be seen in Figure 5 for the OASIS‐3 and Figures S1 and S2 for the SIMON dataset.

**TABLE 2:** Mean reproducibility errors

	Global mean thickness		ROI‐average	
	OASIS‐3	SIMON	OASIS‐3	SIMON
**FreeSurfer**	0.481%	0.674%	1.402%	1.624%
**DL+DiReCT**	0.492%	0.561%	1.287%	1.319%
**ANTs**	2.601%	1.517%	3.149%	2.533%
DL+DiReCT (FS parc.)	0.497%	0.589%	1.358%	1.449%

*Note:* The last row shows supplementary results when using FreeSurfer parcellations.

### 3.3 Annual atrophy rates

Longitudinal annual atrophy rates for global mean cortical thickness are listed in Table 3 for the three sub‐cohorts: healthy controls (*n* = 368 subjects), CDR 0.5 (*n* = 31) and Dementia (*n* = 7). Mean and standard deviation were consistently lowest in FreeSurfer, slightly but statistically not significantly higher with DL+DiReCT and substantially higher with ANTs, which is also visible in Figure S4.

**TABLE 3:** Mean (SD) annual cortical GM atrophy rates in mm/year for the longitudinal data in OASIS‐3

	HC	CDR = 0.5	Dementia (CDR > = 1)
**FreeSurfer**	−0.00711 (±0.01164)	−0.02290 (±0.02871)	−0.02020 (±0.03076)
**DL+DiReCT**	−0.00815 (±0.01444)	−0.02545 (±0.03260)	−0.02290 (±0.04069)
**ANTs**	−0.02039 (±0.06500)*	−0.04383 (±0.06308)*	−0.04983 (±0.08488)
DL+DiReCT (FS parc.)	−0.00820 (±0.01457)	−0.02538 (±0.03287)	−0.02309 (±0.04130)

*Note:* The last row shows supplementary results when using FreeSurfer parcellations. Entries marked with ‘*’ are statistically significant (paired *t*‐test, *p* < .05) different from FreeSurfer.

**FIGURE 6:**
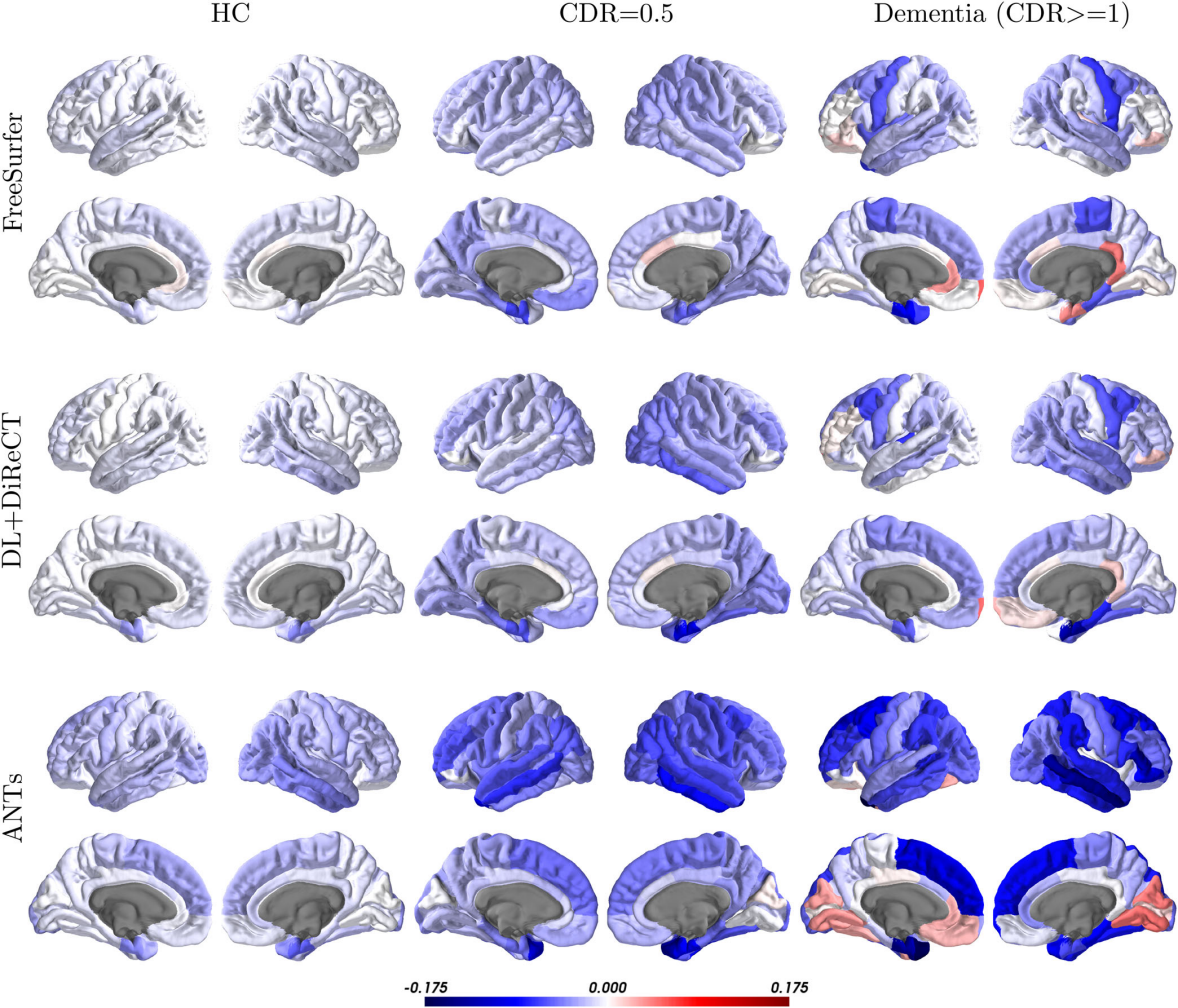
Color‐coded annual atrophy rates in mm/year of the ROI‐wise average cortical thicknesses evaluated on the OASIS‐3 samples

Regional atrophy rates are depicted in Figure 6. The most pronounced atrophy rates in the dementia cohort were observed in the left entorhinal cortex (−0.089 mm/year) for FreeSurfer, in the right entorhinal cortex (−0.129 mm/year) for DL+DiReCT, and in the left temporal pole (−0.175 mm/year) for ANTs. The corresponding Figure S5 shows these changes in relation to the global atrophy rate to make regional differences better visible. An additional cross‐sectional analysis is depicted in Figure S3.

### 3.4 Group‐wise differences

**FIGURE 7:**
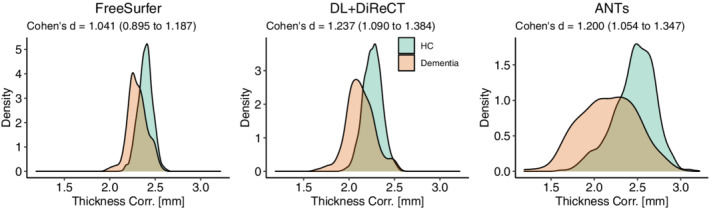
Kernel density plots of the global mean thickness, corrected for brain size and age, depicting effect‐size (Cohen's *d* reported in the subtitle) between healthy controls (HC) and dementia

Group‐wise differences between HC and dementia for the global mean thicknesses corrected for brain size and age were largest with DL+DiReCT (Cohen's *d* = 1.237, CI_95%_ = 1.090 − 1.384) followed by ANTs (*d* = 1.200, CI_95%_ = 1.054 − 1.347), and FreeSurfer (*d* = 1.041, CI_95%_ = 0.895 − 1.187), as depicted in Figure 7.

## 4 DISCUSSIONS AND CONCLUSION

We propose DL+DiReCT, combining a deep learning‐based neuroanatomy segmentation with diffeomorphic registration to measure subtle changes in cortical thickness from MRI. Our experiments suggest that the method is potentially more sensitive than, and comparably robust to, surface‐based measurement as used by FreeSurfer. In the absence of a gold‐standard ground truth for cortical thickness measures, we assessed the accuracy on two independent datasets by indirect means: correlation with FreeSurfer, robustness with a large number of re‐scans, cross‐sectional and longitudinal gray‐matter atrophy rates, and sensitivity to detect group‐wise differences of healthy controls compared to individuals with dementia. We have used FreeSurfer as the *silver‐standard* ground truth in this study since we consider it the most established tool in the field. However, these surface‐based measures are not meant to be replicated as close as possible but serve as a good reference point to complement outcome‐based evaluations like robustness, effect size, and plausibility of observations in the light of underlying biological processes.

In agreement with the large‐scale evaluation by Tustison et al. (2014) on OASIS‐3, we found almost identical values of the Pearson correlation coefficients with FreeSurfer for ANTs (*r* = .45). We also observed the higher sensitivity of ANTs to detect age‐related gray‐matter atrophy. However, we conclude this additional sensitivity comes at the cost of lower robustness (mean reproducibility error of 3.1%, that is, more than twice the value of FreeSurfer) due to inferior atlas‐based segmentation. By replacing the atlas‐based segmentation with a deep learning‐based model in DL+DiReCT, correlation with Freesurfer thickness measures can be significantly increased (*r* = .72), while also achieving robustness (mean reproducibility error 1.3%) comparable to FreeSurfer (mean reproducibility error 1.4%). These observations are in concordance with the study of Clarkson et al. (2011) which also reports generally lower robustness for ANTs and higher standard deviations for longitudinal atrophy rates compared to FreeSurfer.

The sensitivity and robustness of DL+DiReCT permits the measurement of (cf., Table 3) subtle longitudinal annual atrophy rates of 0.008 mm/year in the group of healthy controls (*n* = 368 samples) and 0.025 mm/year in the CDR = 0.5 cohort (*n* = 31). These absolute values show a remarkably high agreement with FreeSurfer (no statistically significant difference), and it is worth noting once again that the surface‐based cortical thickness measures of FreeSurfer were not used in any way in the training process of the DL+DiReCT method (only the segmentation from FreeSurfer was used during training).

For DL+DiReCT we observed (cf., Figure 6) regional patterns for the CDR = 0.5 and dementia cohort similar to what has been reported by others: Higher atrophy rates in the medial and lateral temporal lobe (Fennema‐Notestine et al., 2009; Fujishima et al., 2014; Lerch et al., 2005; Thompson et al., 2003), most pronounced in the entorhinal cortex (Lerch et al., 2005; Thompson et al., 2003), supporting the hypothesis of disease onset in these regions (Atiya, Hyman, Albert, & Killiany, 2003; Braak & Braak, 1991; Thompson et al., 2003). The results also suggest relative sparing of the somatosensory cortex (Fennema‐Notestine et al., 2009; Frisoni et al., 2010; Lerch et al., 2005; Thompson et al., 2003). Putative increase of thickness in the cuneus and lingual gyrus in the dementia cohort reported by ANTs are likely due to lower robustness of the method as well as the reported increasing thickness in the right entorhinal cortex by FreeSurfer.

DL+DiReCT runs in about 1 hour, producing segmentations and a volumetric thickness map (see Figure 1) that allows a visual inspection of the result by humans, partially opening the black‐box of fast deep learning‐based morphometry methods (Rebsamen, Suter, Wiest, Reyes, & Rummel, 2020).

### 4.1 Limitations

Minimally preprocessed (skull‐stripping and resampling into FreeSurfer space) input data was used to facilitate a direct comparison of results using FreeSurfer parcellations. As the original data was already in 1 mm iso‐voxel resolution, we are confident that this does not significantly influence the results. By using the same skull‐stripping for all methods, we can avoid side effects from different brain extraction techniques.

We have not tried other deep learning‐based segmentation methods, since finding the best network architecture was not the focus of this study. Other methods likely yield similar results if the model achieves high accuracy even with a large number of labels required for the cortex parcellation.

The number of longitudinal samples in the confirmed dementia cohort was low (*n* = 7), limiting the power of statistical tests. However, the plausibility of the results is supported by the observed atrophy patterns suggesting a trajectory of the disease known from literature and the additional cross‐sectional analysis (*n* = 209). For specific analysis of longitudinal changes, the robustness might be further improved with the dedicated longitudinal pipeline available in FreeSurfer (Reuter, Schmansky, Rosas, & Fischl, 2012) and ANTs (Tustison et al., 2019), none of which was used in the current study to facilitate a direct comparison of the methods.

### 4.2 Outlook

We intend to continue optimizing the proposed method. Namely investigating whether applying DiReCT to segmentations with higher spatial resolution, either from 7T imaging or via super‐resolution, increases the sensitivity while preserving the good robustness. While DL+DiReCT is already substantially faster than the frequently used methods FreeSurfer and ANTs, the computationally most expensive step of the current solution is the 3D registration: replacement by a (separate) deep learning‐based registration is expected to reduce the total runtime to a few minutes (Dalca et al., 2019; Mok, 2020). Additionally, measures for the cortical curvature and surface area could be derived from the diffeomorphic model. A dedicated longitudinal mode for DL+DiReCT is conceivable by deriving thickness changes from registering the inner and outer surface of two or more time points, which would also allow a direct comparison to FreeSurfer's longitudinal pipeline. The current deep‐learning model is trained to predict 96 different labels. Further increasing the number of labels with a more fine‐grained atlas like Destrieux (Destrieux, Fischl, Dale, & Halgren, 2010) with its 74 parcellations per hemisphere would be of interest as it is used in many morphometry studies, and might reveal where the model's segmentation performance starts deteriorating. Future work will also extend the evaluation to further applications where subtle changes of cortical thickness are of high clinical interest.

## DATA AVAILABILITY STATEMENT

The data that support the findings of this study are openly available at https://central.xnat.org/ for OASIS‐3 and at https://doi.org/10.15387/fcp_indi.retro.simon for SIMON.

## Supporting information


**Appendix** **S1:** Supplementary Material
