# Supplementary material for: Direct cortical thickness estimation using deep learning‐based anatomy segmentation and cortex parcellation
Source: Hum Brain Mapp. 2020 Aug 12;41(17):4804–14. doi: 10.1002/hbm.25159 (PMC7643371; doi:10.1002/hbm.25159)

Supplementary Materials for Direct Cortical Thickness Estimation using Deep Learning-based Anatomy Segmentation

Michael Rebsamen[1,2], Christian Rummel[1], Mauricio Reyes[3,4], Roland Wiest[1], and Richard McKinley[1]

[1]Support Center for Advanced Neuroimaging (SCAN), University Institute of Diagnostic and Interventional Neuroradiology, University of Bern, Inselspital, Bern University Hospital, Bern, Switzerland
[2]Graduate School for Cellular and Biomedical Sciences, University of Bern, Bern, Switzerland
[3]Insel Data Science Center, Inselspital, Bern University Hospital, Bern, Switzerland
[4]ARTORG Center for Biomedical Research, University of Bern, Bern, Switzerland

Contents

1 Anatomy Segmentation 1

2 Robustness SIMON Dataset 2

3 Cross-sectional OASIS-3 Data 3

4 Longitudinal OASIS-3 Data 4

5 Regional Cortical Thickness Plots 6

1 Anatomy Segmentation

	**White matter**		**Cortical gray matter**		**Amygdala**		**Hippocampus**	
	lh	rh	lh	rh	lh	rh	lh	rh
OASIS-3	0.970 (0.95-0.99)	0.970 (0.95-0.99)	0.955 (0.93-0.98)	0.953 (0.92-0.98)	0.855 (0.78-0.92)	0.859 (0.78-0.93)	0.901 (0.85-0.94)	0.906 (0.87-0.94)
SIMON	0.976 (0.97-0.98)	0.976 (0.97-0.98)	0.963 (0.95-0.98)	0.960 (0.94-0.98)	0.868 (0.82-0.92)	0.864 (0.83-0.90)	0.903 (0.88-0.92)	0.884 (0.86-0.91)

Table S1: Performance of the segmentation in terms of median Dice coefficients (1.5$\times$ IQR) against FreeSurfer for the relevant structures on the two datasets. lh: left hemisphere, rh: right hemisphere.

2 Robustness SIMON Dataset

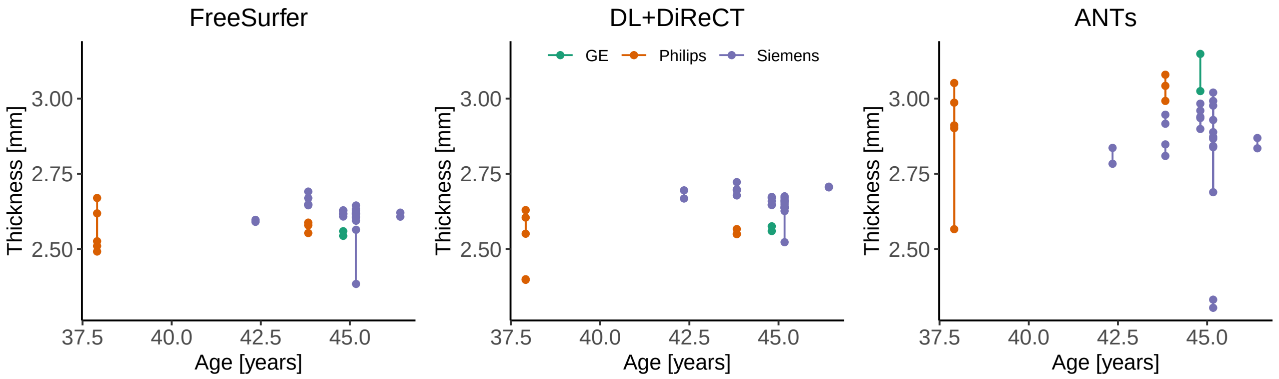

Figure S1: Global mean thicknesses for the re-scans in the SIMON dataset. Connected measures are from the same session. Colors indicate scanner manufacturer.

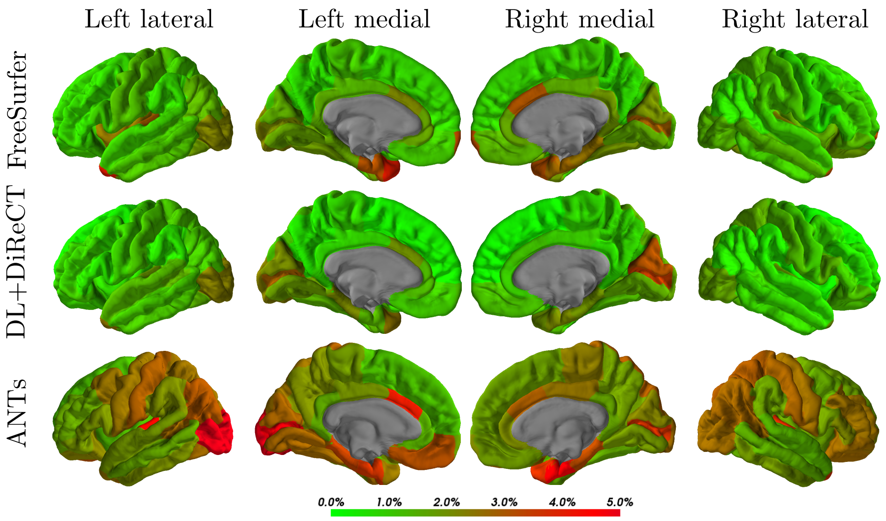

Figure S2: Color-coded reproducibility errors of the ROI-wise average cortical thicknesses evaluated on the SIMON samples.

3 Cross-sectional OASIS-3 Data

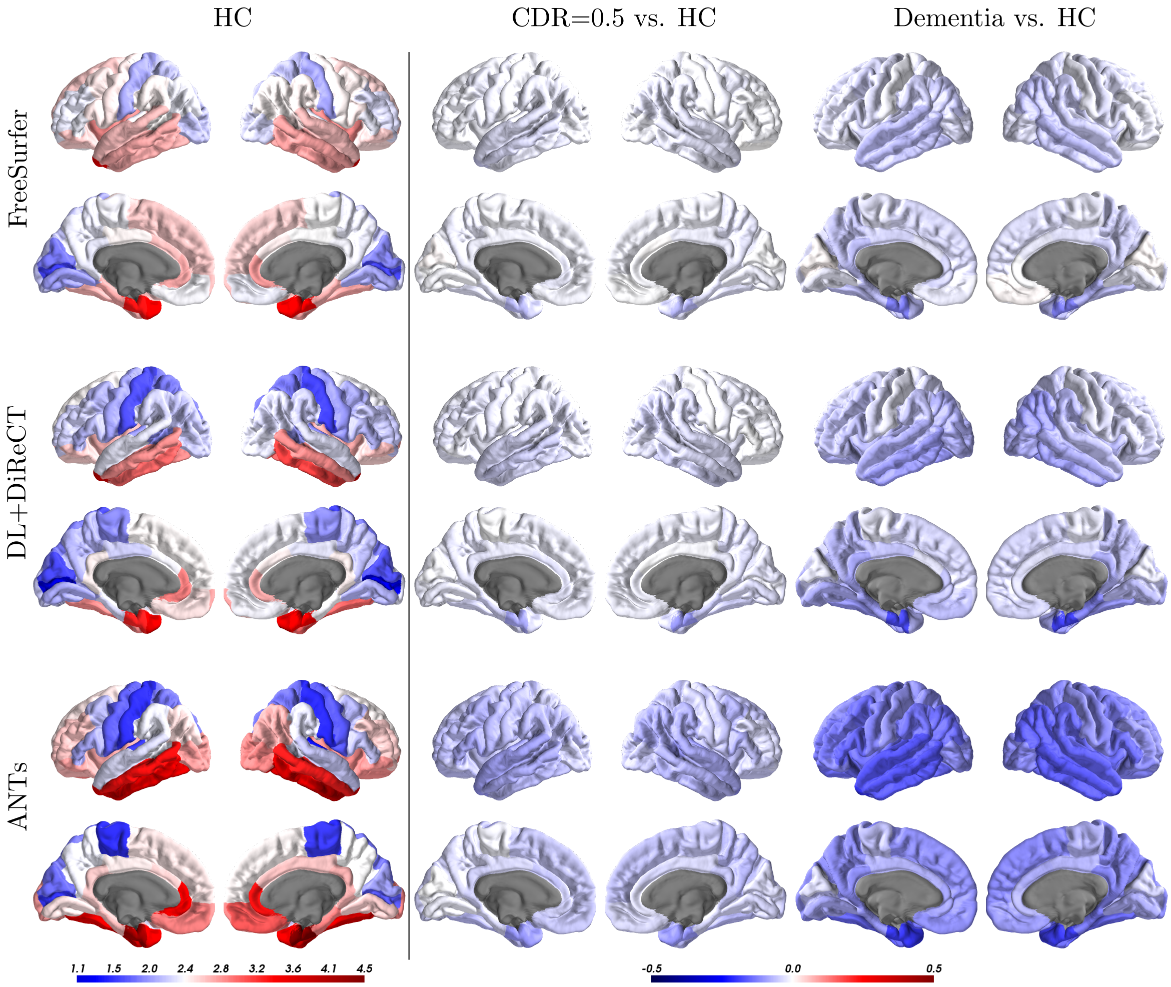

Figure S3: Left: Cross-sectional mean cortical thickness per region for healthy controls (HC). The color scale is centered around the global mean thickness of all three methods (2.37mm). Right: Relative change of cross-sectional mean cortical thicknesses compared to the HC cohort. Cortical thicknesses are corrected for brain size and age.

4 Longitudinal OASIS-3 Data

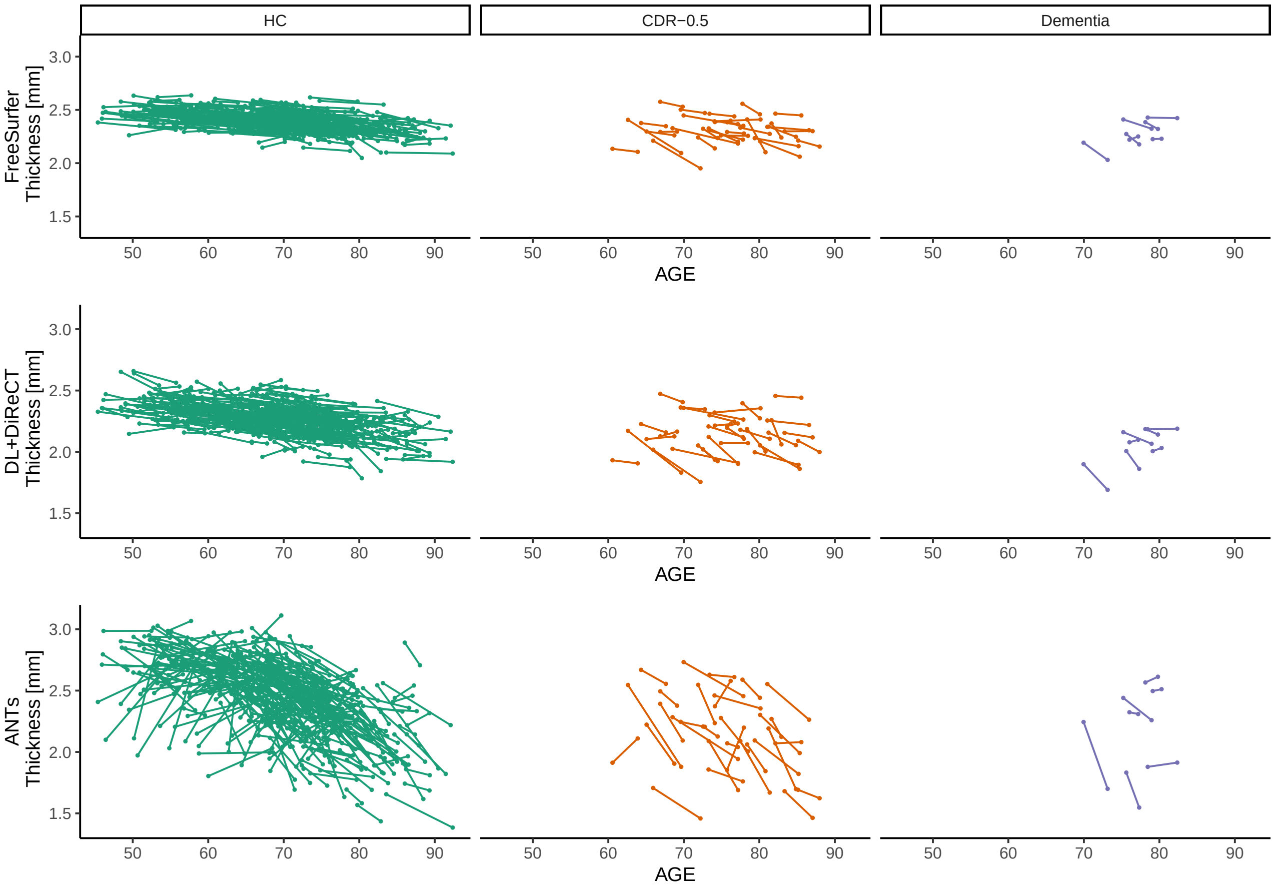

Figure S4: Visualization of OASIS-3 longitudinal data for the global mean thickness. Lines connect the first and last scan from the same subject.

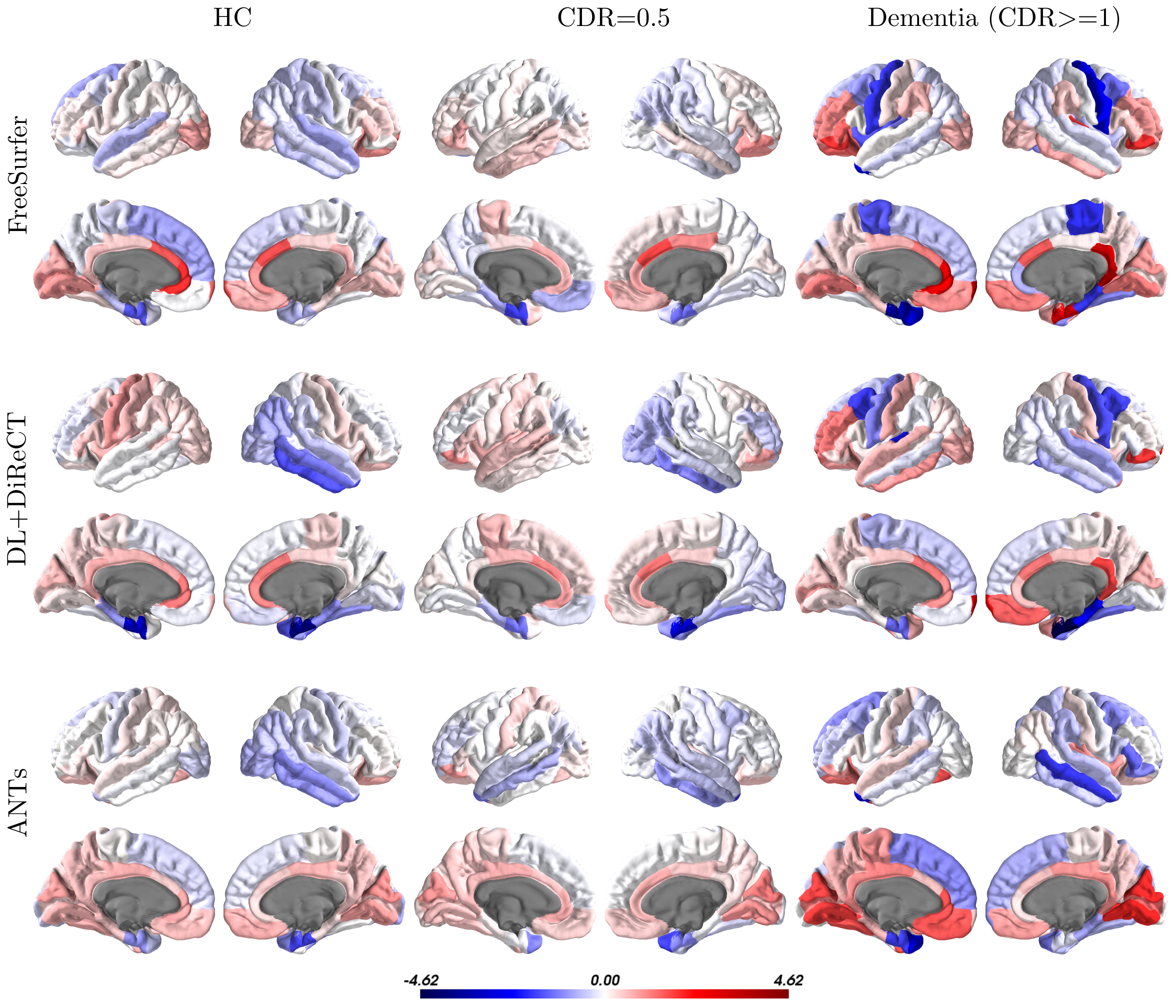

Figure S5: ROI-wise atrophy rates **relative** to the global atrophy rate evaluated on the OASIS-3 samples. Color codes indicate regions showing more atrophy (blue), about the same (white), or less (red) as the global mean atrophy rate.

5 Regional Cortical Thickness Plots

Listed below are plots for all regions of the cortical thickness measures on the OASIS-3 dataset (n=2643). Kernel density plots showing the distribution of the thickness measures of all three methods are followed by thickness measures plotted against age.
FS: FreeSurfer, lh: left hemisphere, rh: right hemisphere.

Banks of the superior temporal sulcus - lh

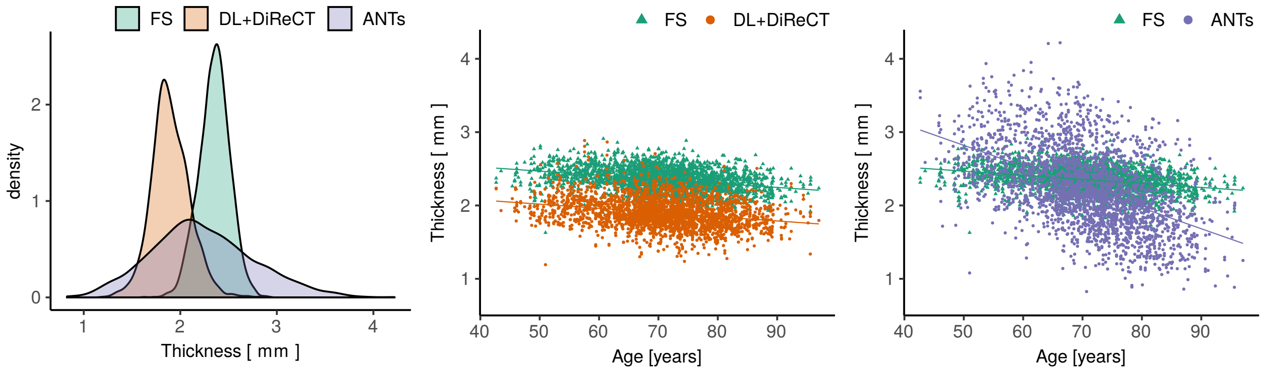

Banks of the superior temporal sulcus - rh

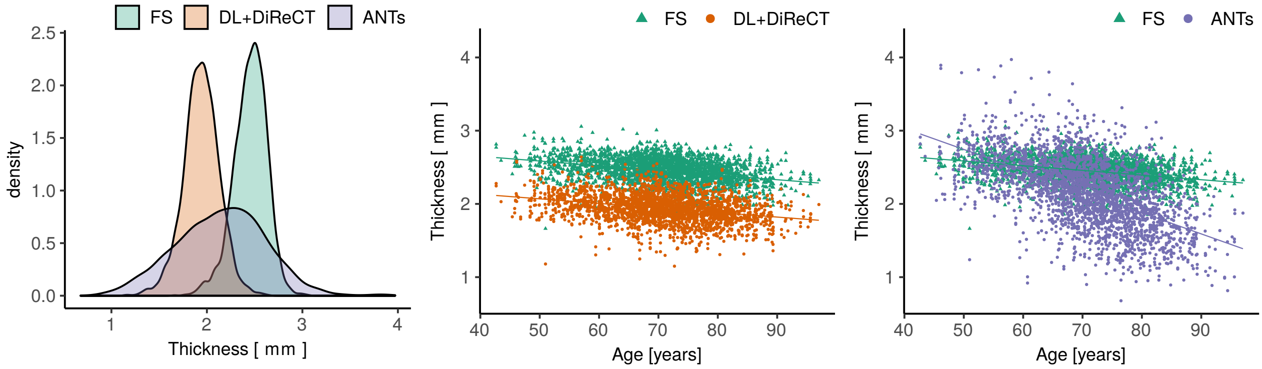

Caudal anterior cingulate cortex - lh

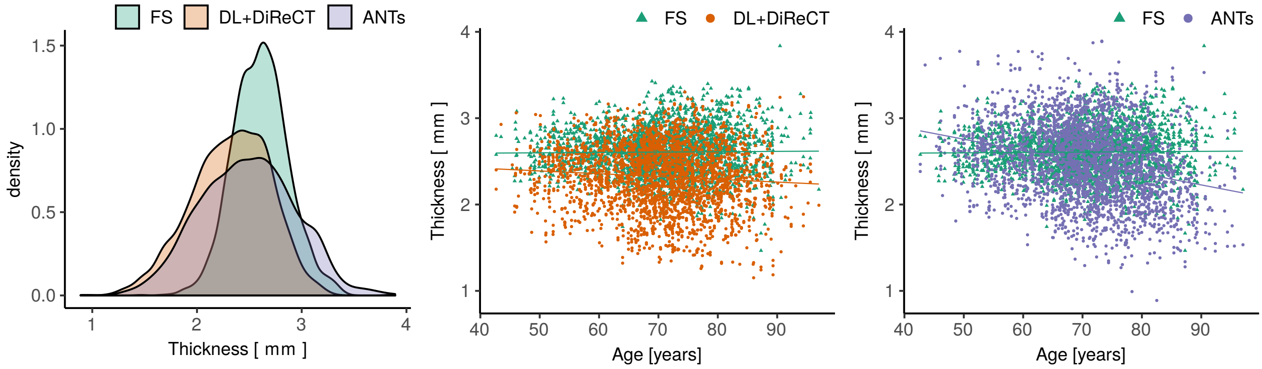

Caudal anterior cingulate cortex - rh

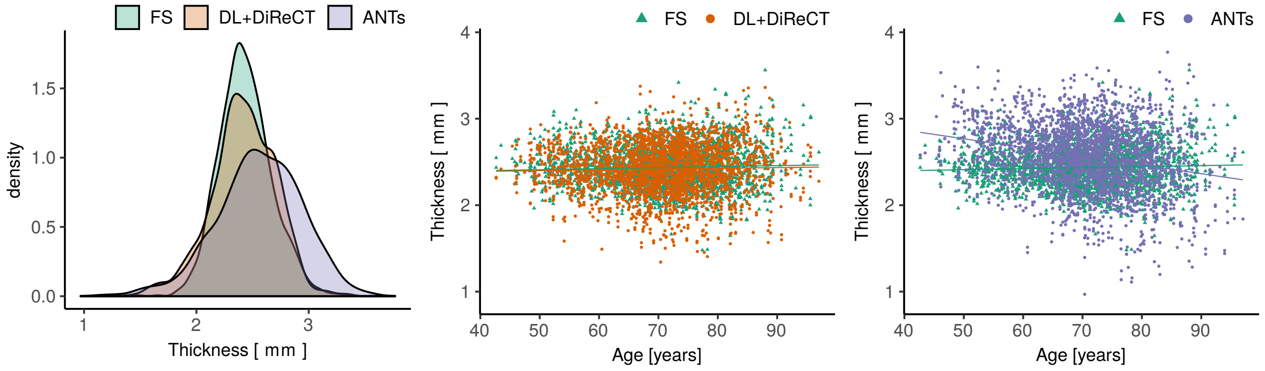

Caudal middle frontal gyrus - lh

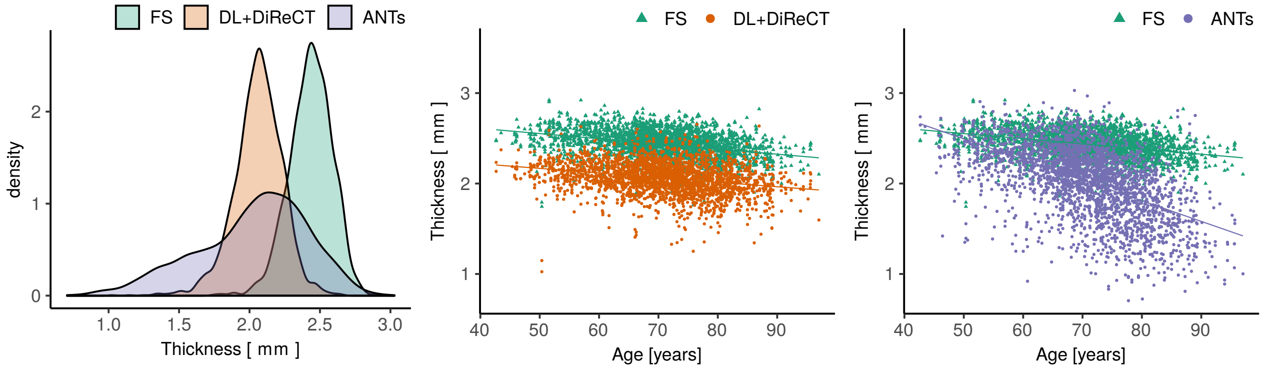

Caudal middle frontal gyrus - rh

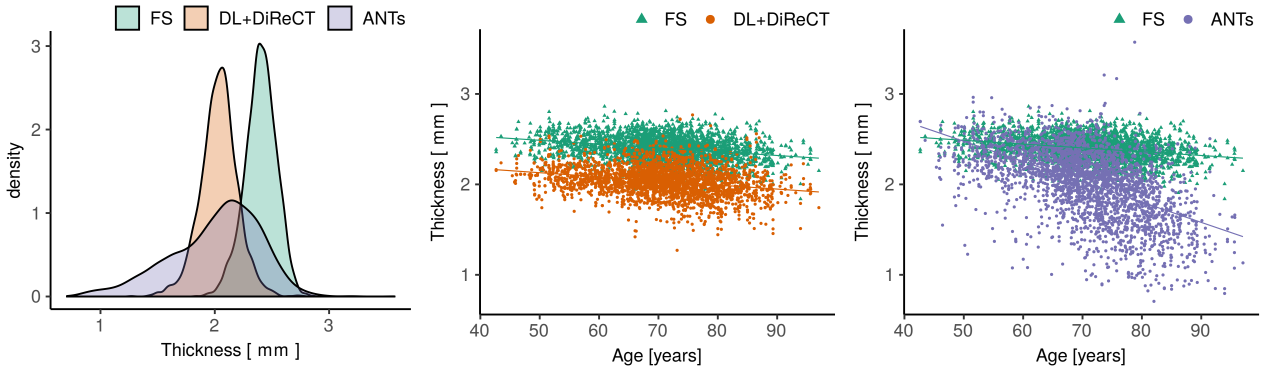

Cuneus - lh

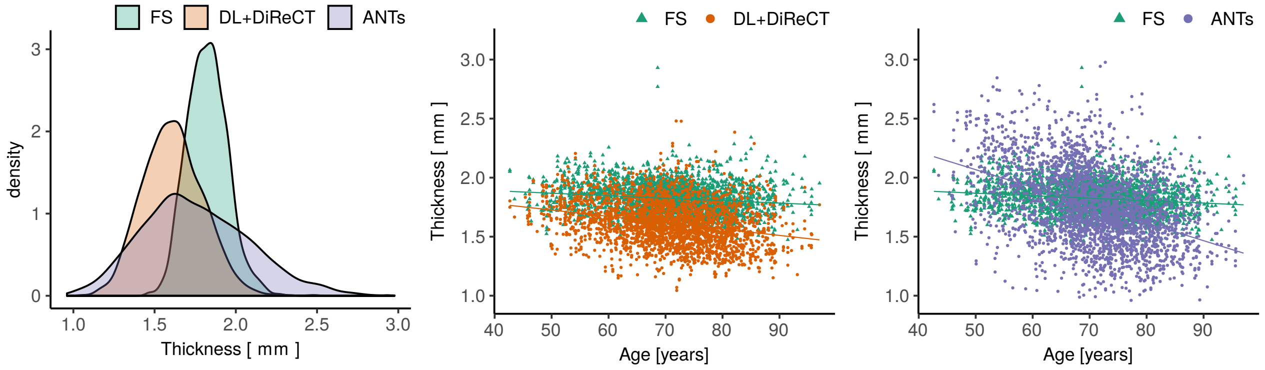

Cuneus - rh

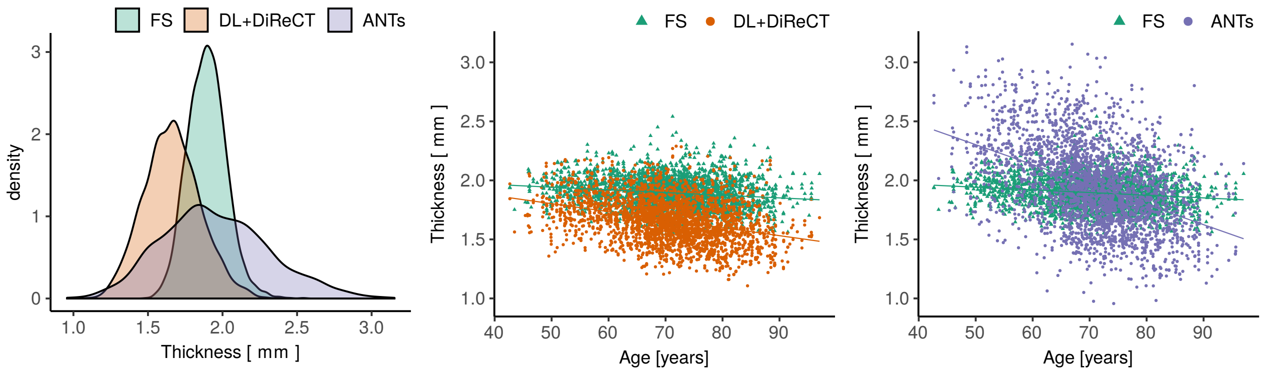

Entorhinal cortex - lh

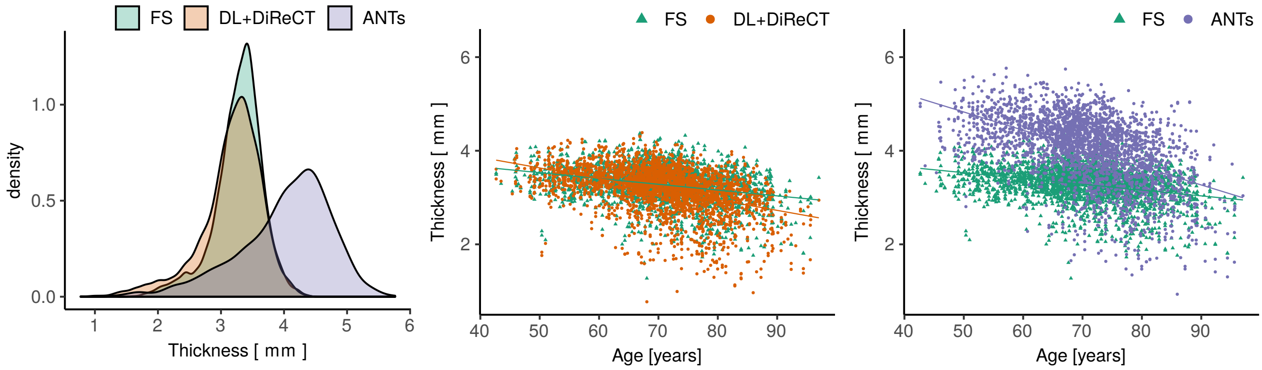

Entorhinal cortex - rh

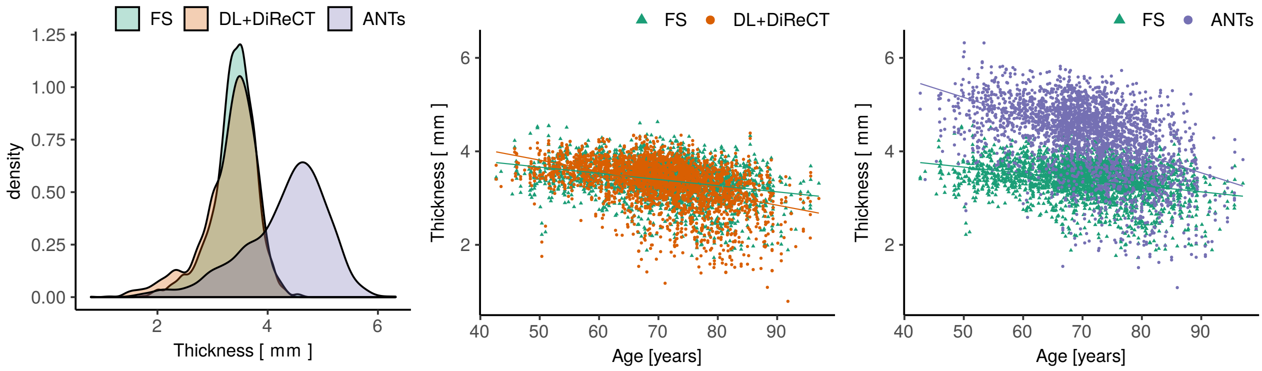

Frontal pole - lh

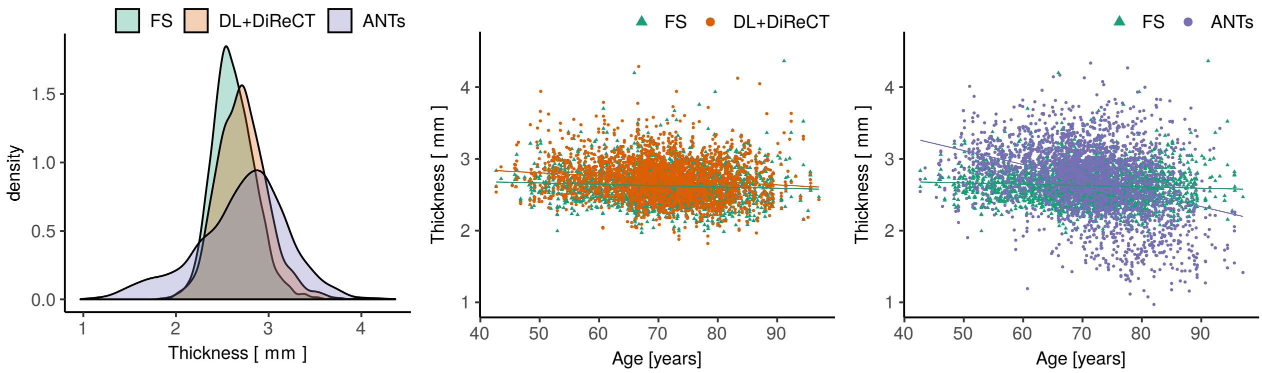

Frontal pole - rh

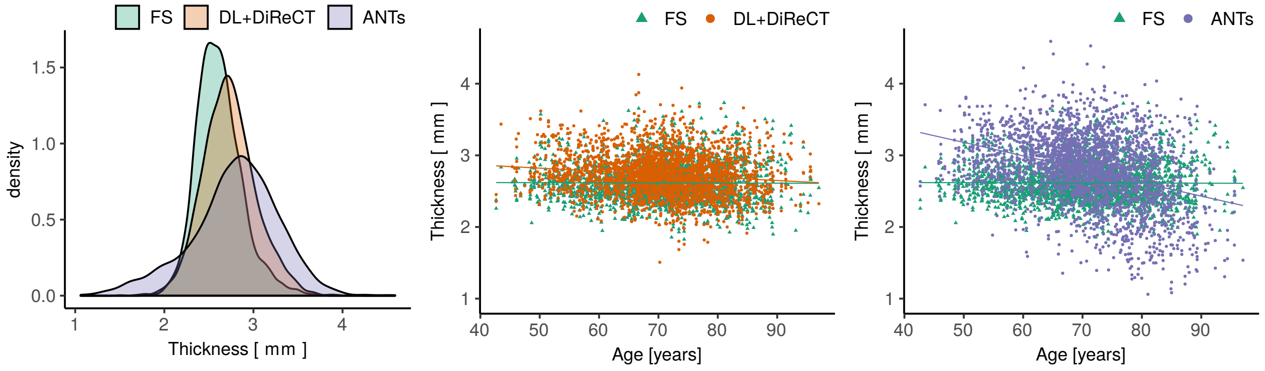

Fusiform gyrus - lh

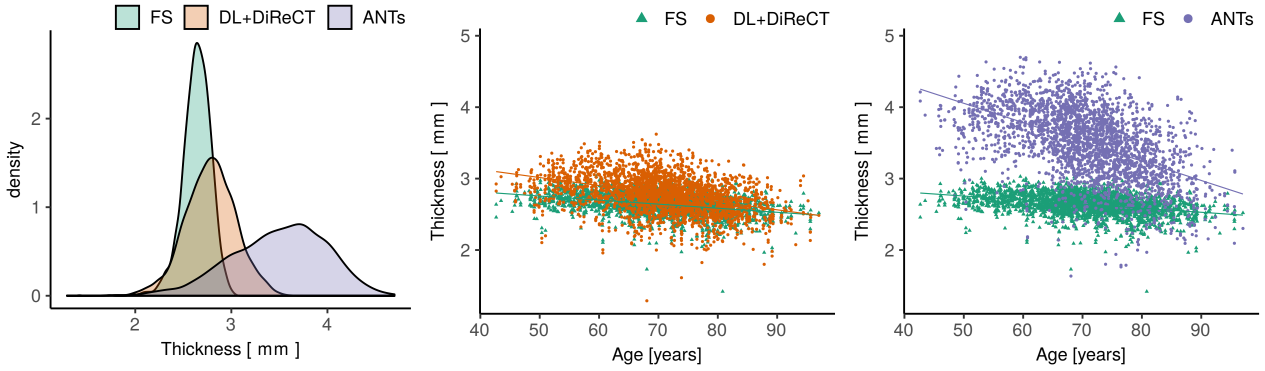

Fusiform gyrus - rh

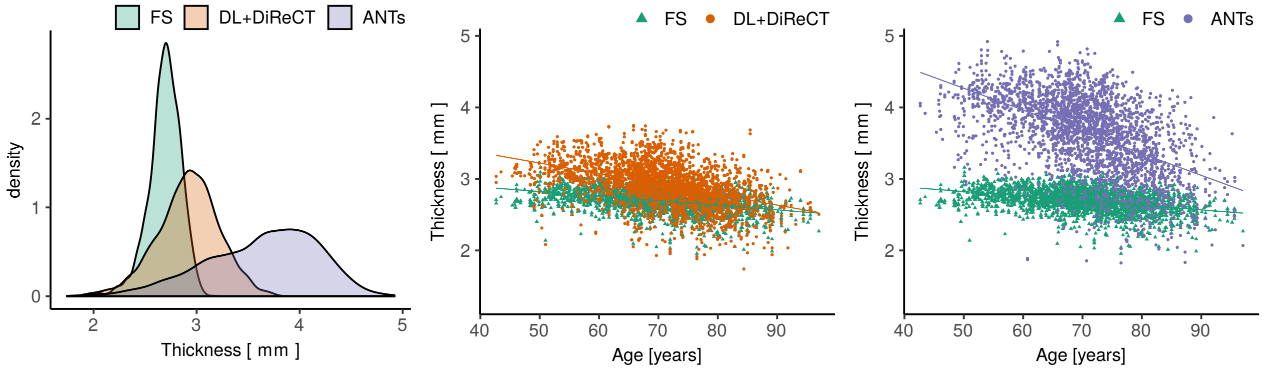

Inferior parietal lobule - lh

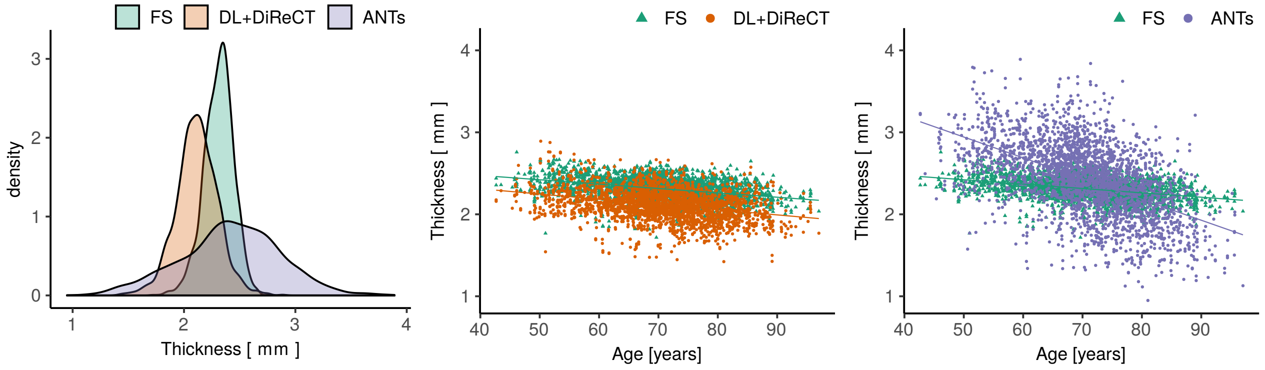

Inferior parietal lobule - rh

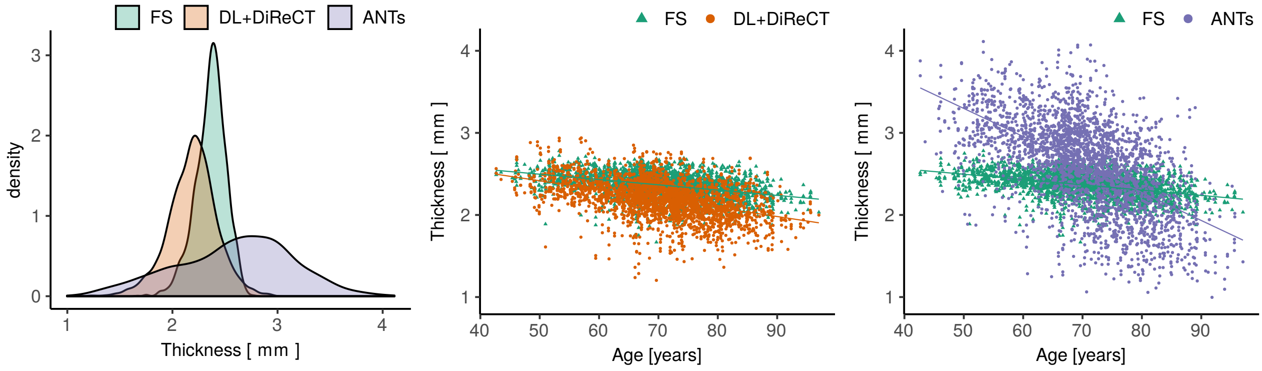

Inferior temporal gyrus - lh

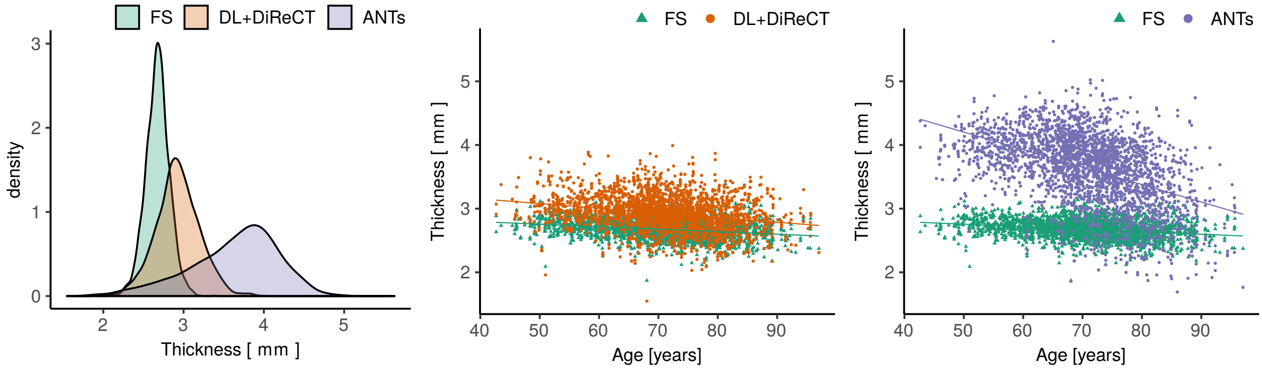

Inferior temporal gyrus - rh

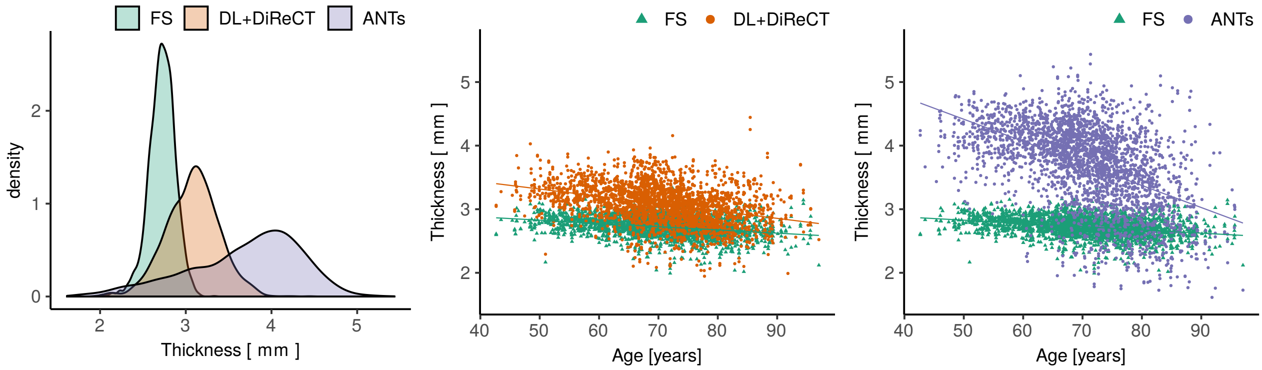

Insular cortex - lh

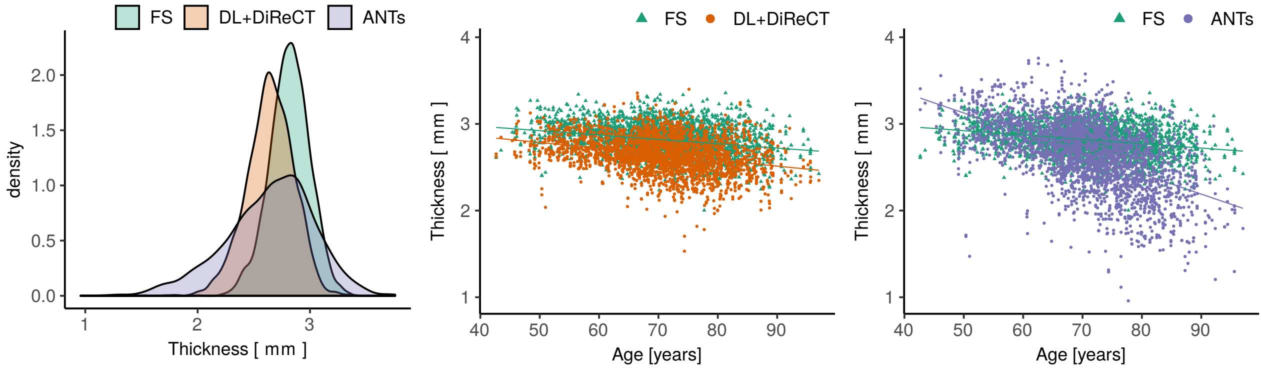

Insular cortex - rh

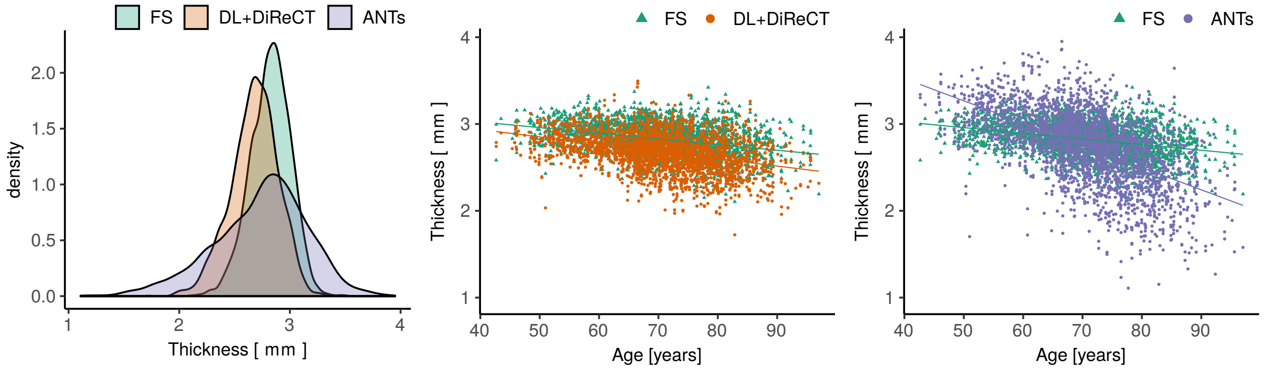

Isthmus of the cingulate cortex - lh

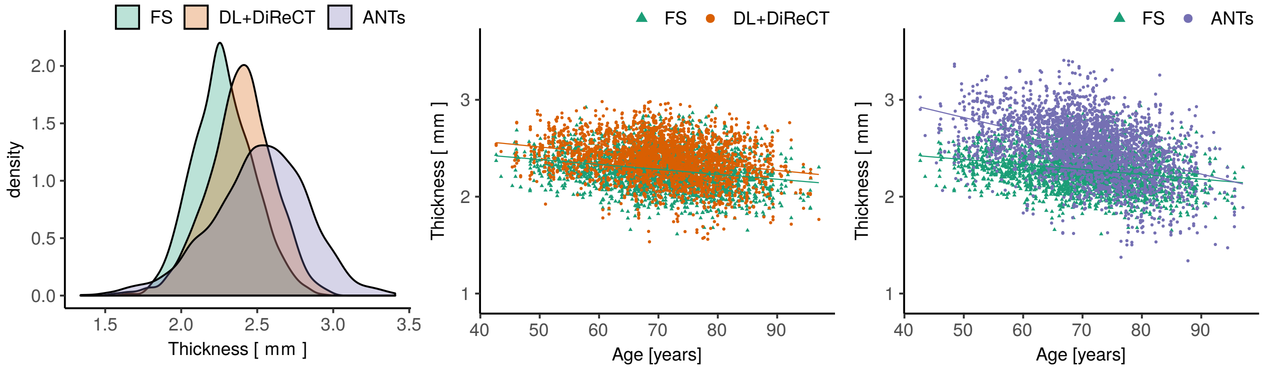

Isthmus of the cingulate cortex - rh

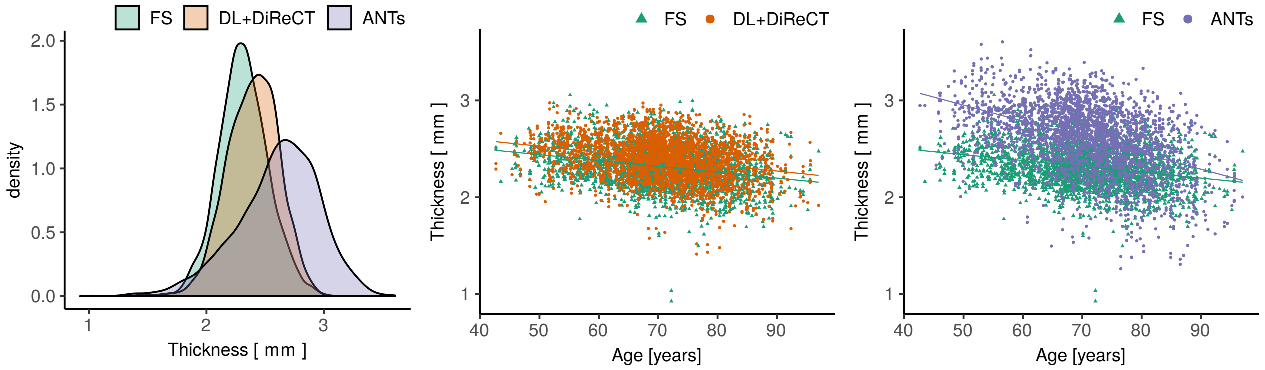

Lateral occipital cortex - lh

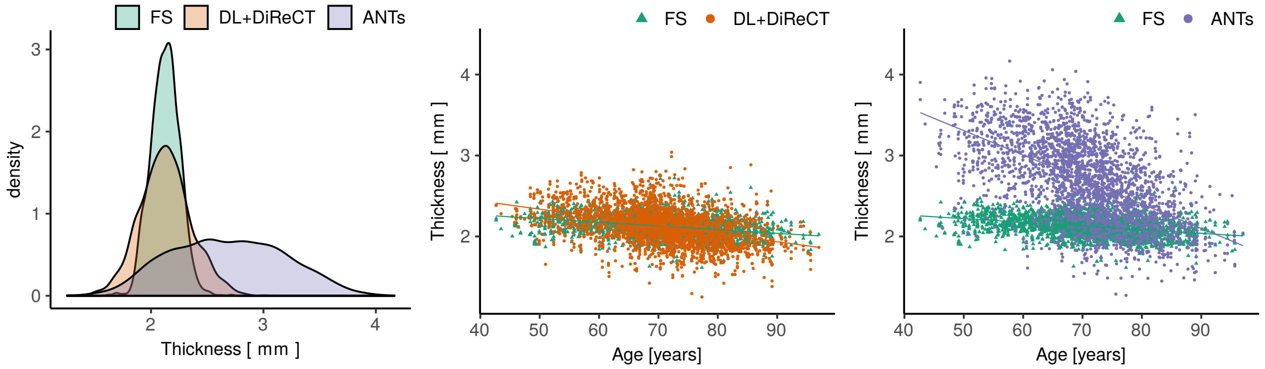

Lateral occipital cortex - rh

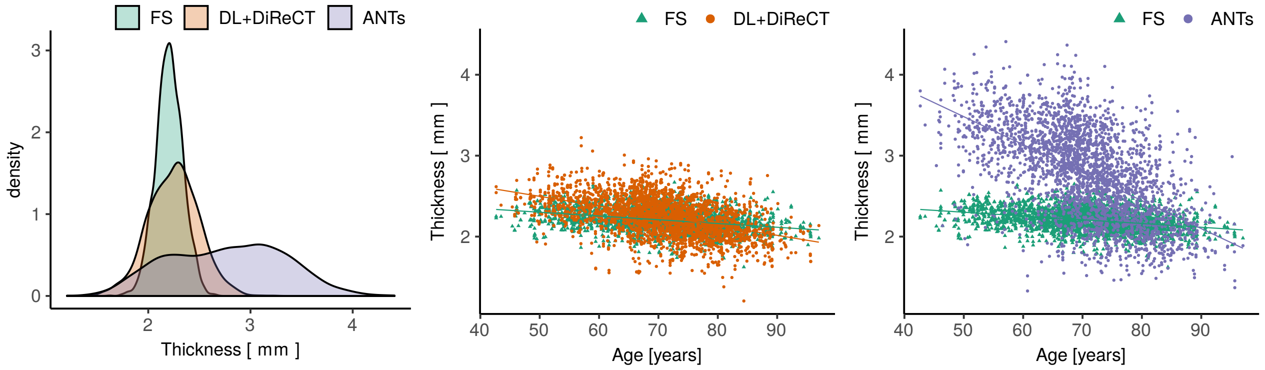

Lateral orbitofrontal cortex - lh

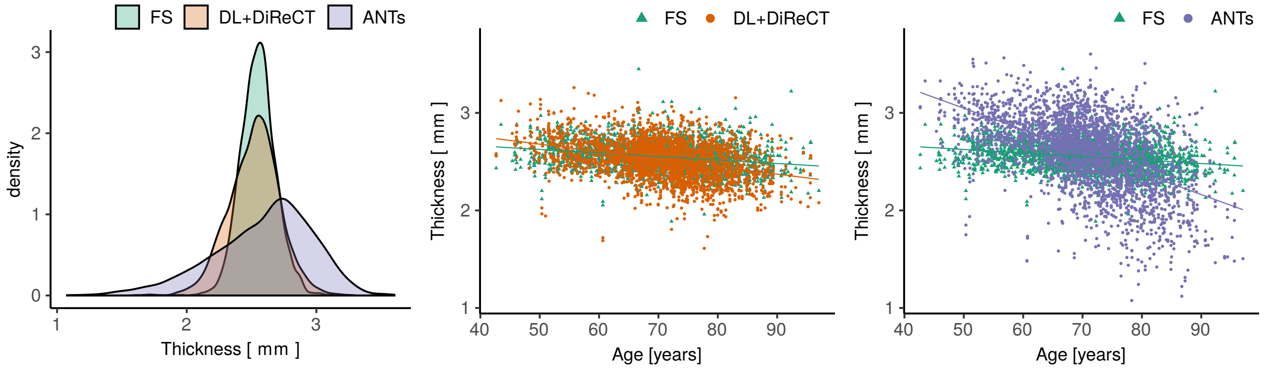

Lateral orbitofrontal cortex - rh

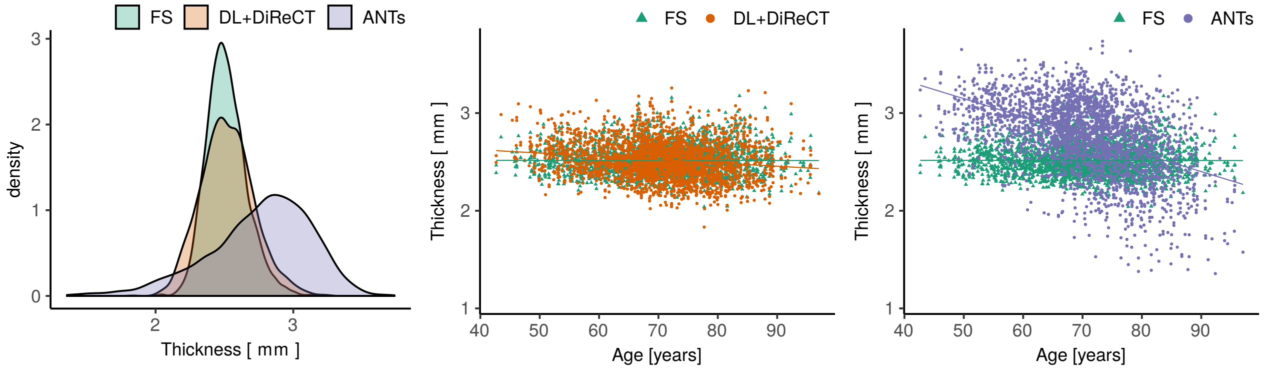

Lingual gyrus - lh

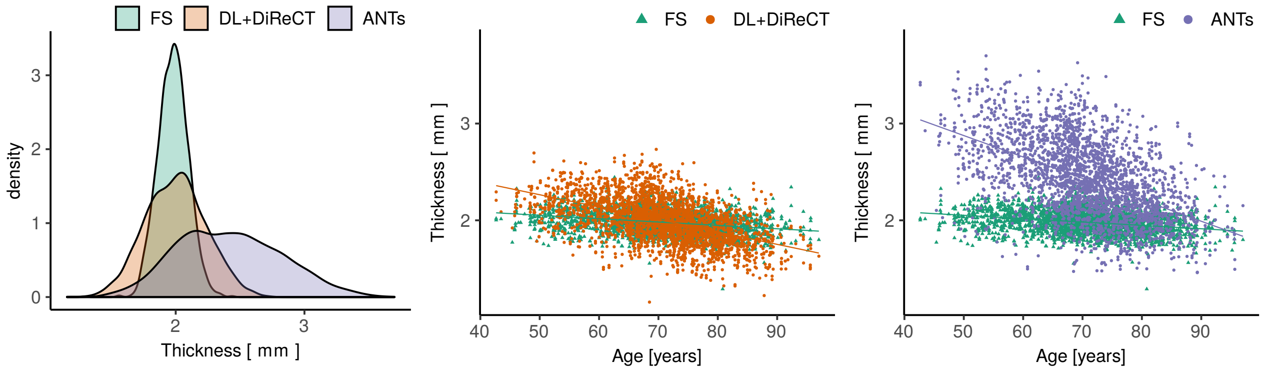

Lingual gyrus - rh

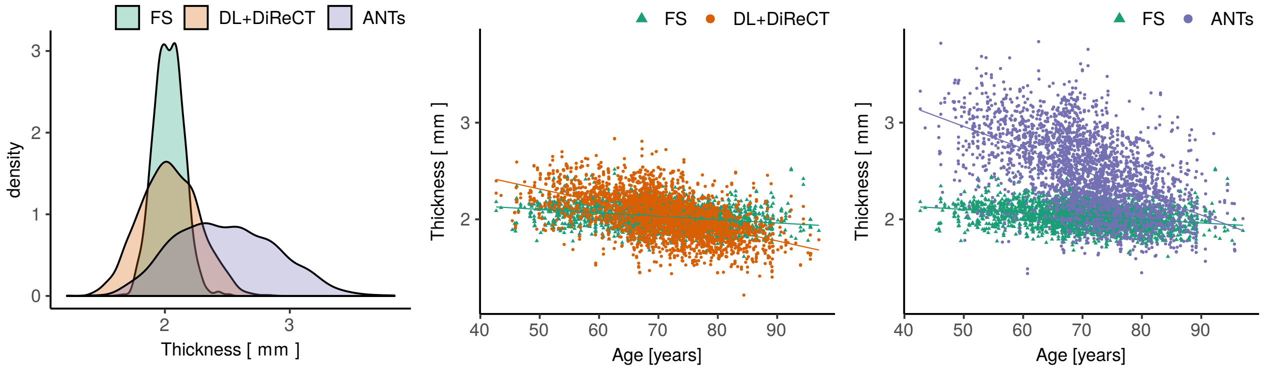

Medial orbitofrontal cortex - lh

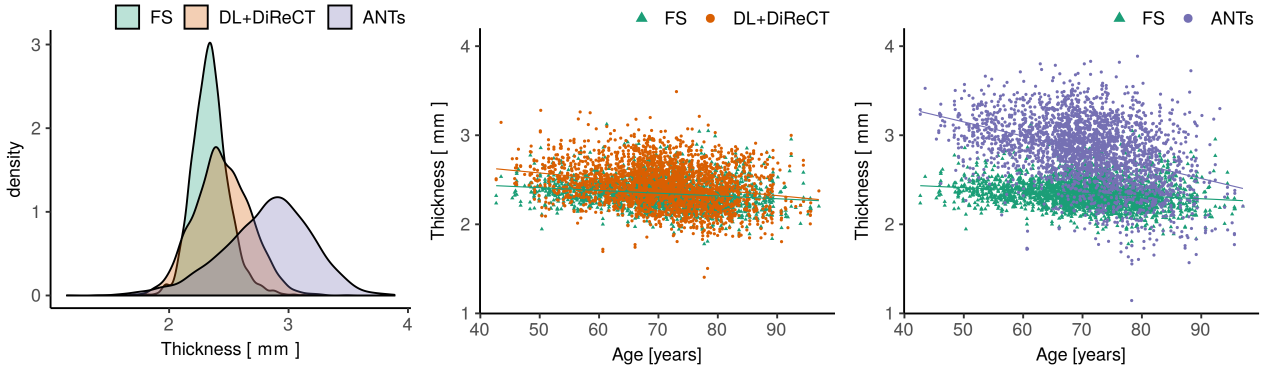

Medial orbitofrontal cortex - rh

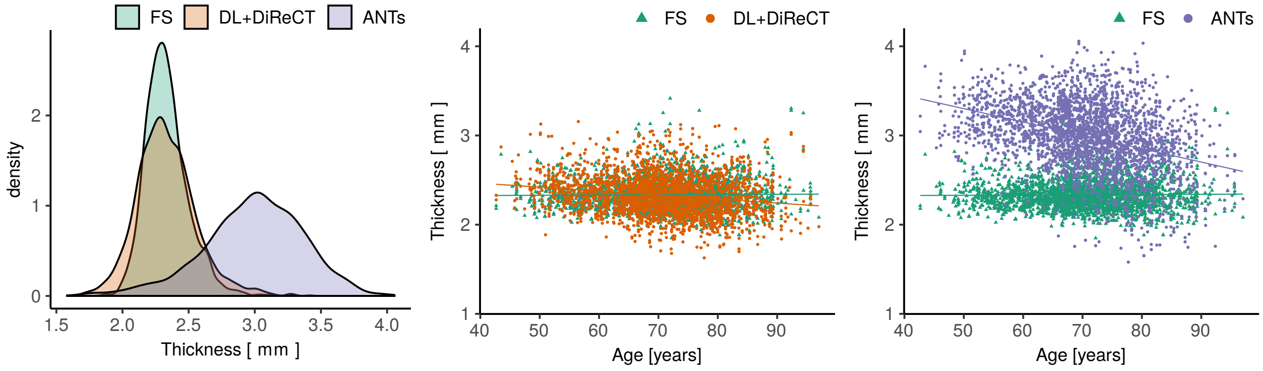

Middle temporal gyrus - lh

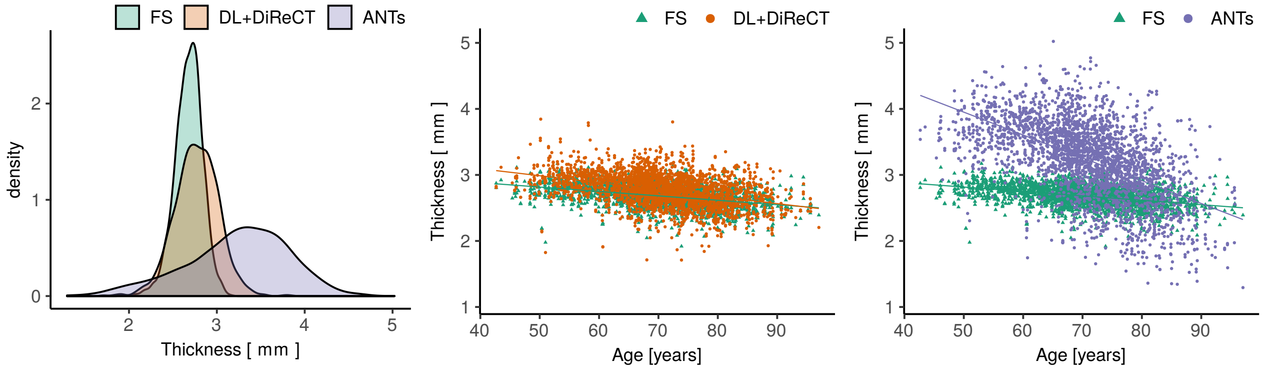

Middle temporal gyrus - rh

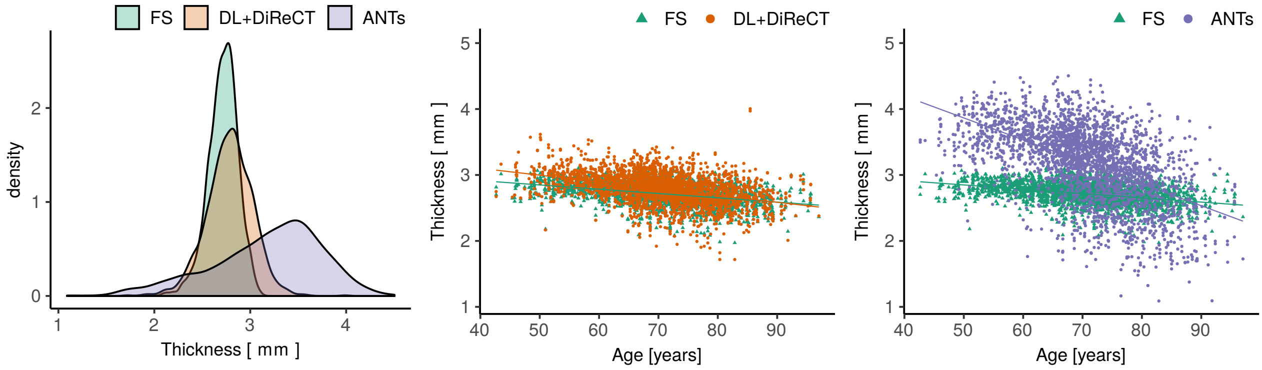

Paracentral lobule - lh

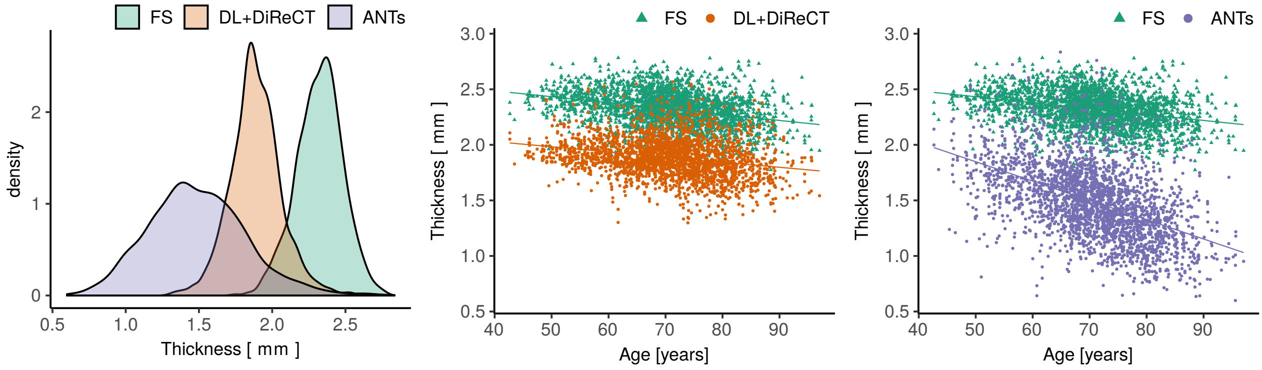

Paracentral lobule - rh

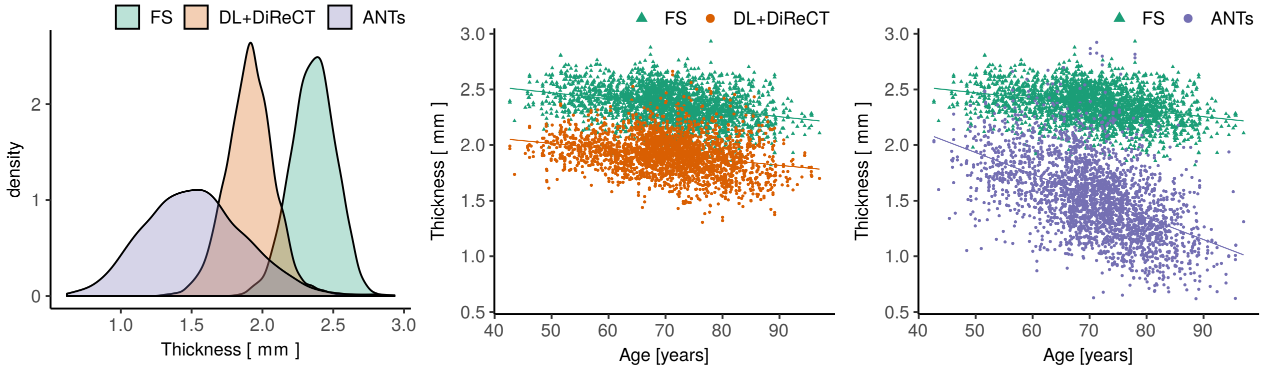

Parahippocampal gyrus - lh

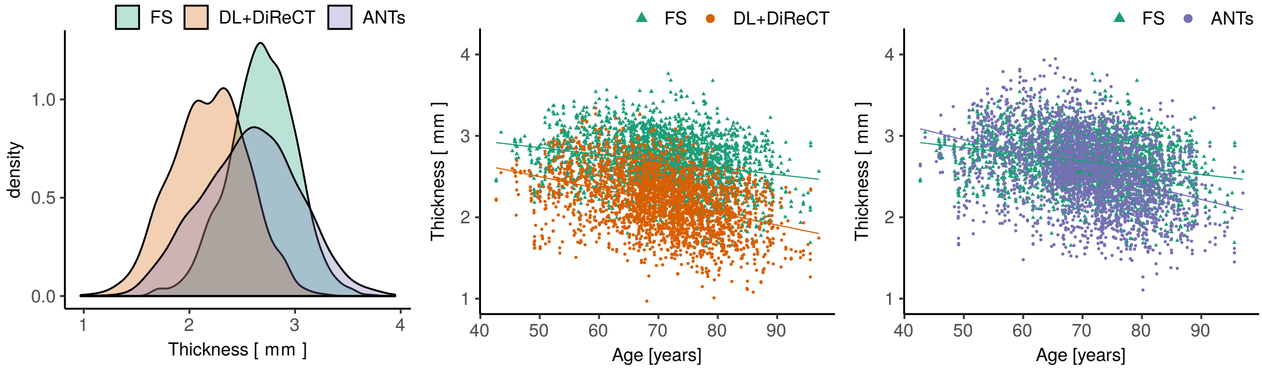

Parahippocampal gyrus - rh

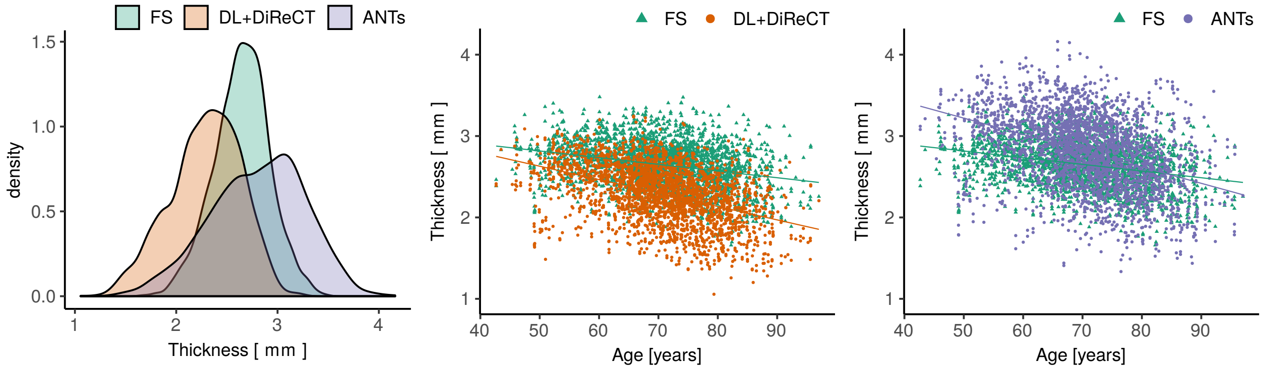

Pars opercularis - lh

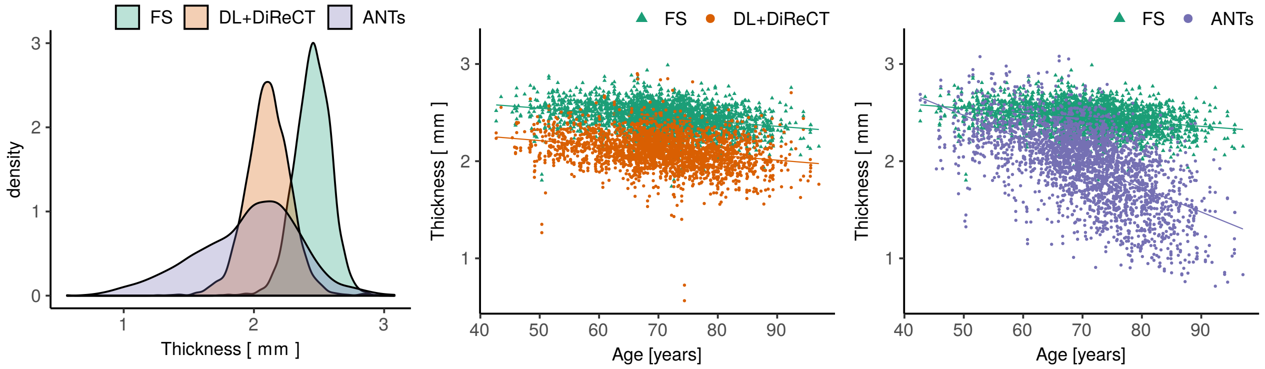

Pars opercularis - rh

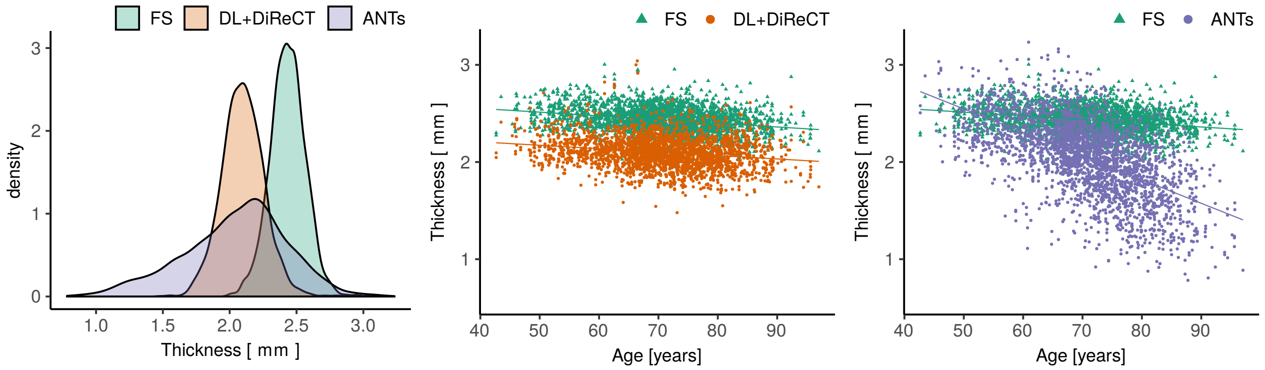

Pars orbitalis - lh

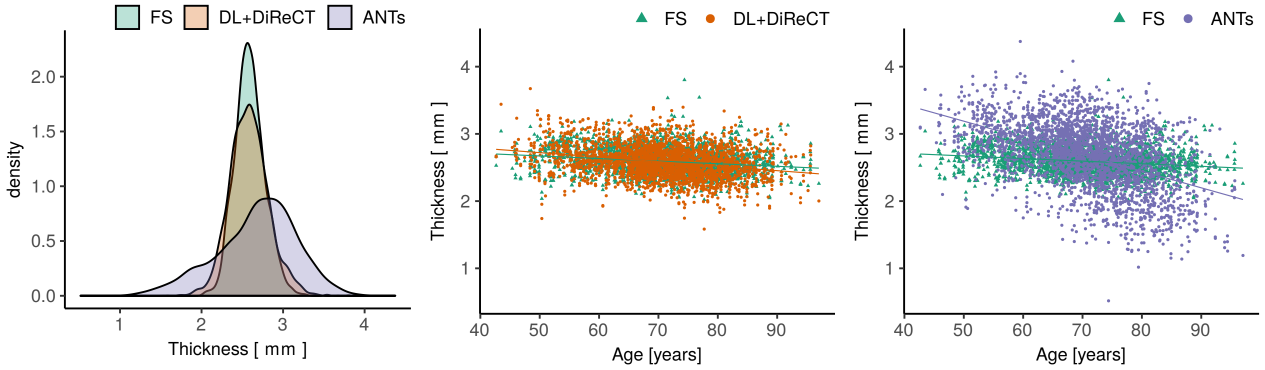

Pars orbitalis - rh

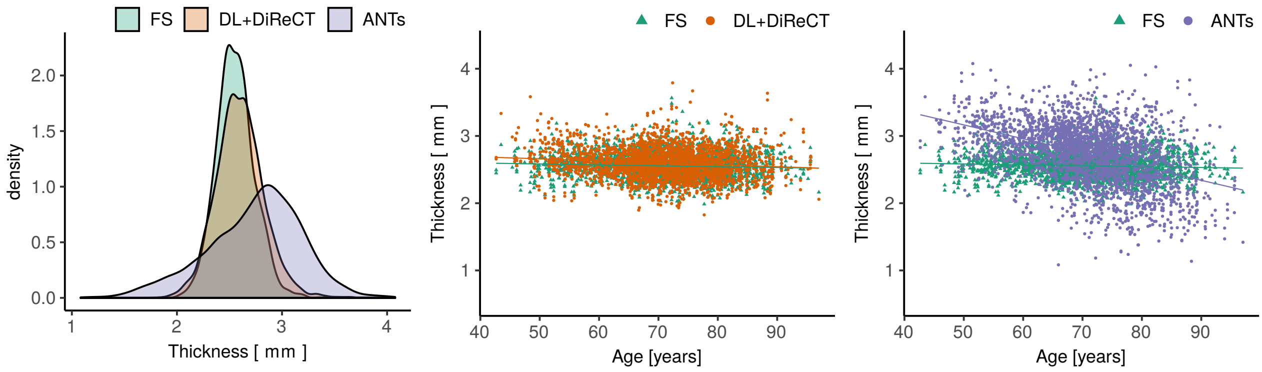

Pars triangularis - lh

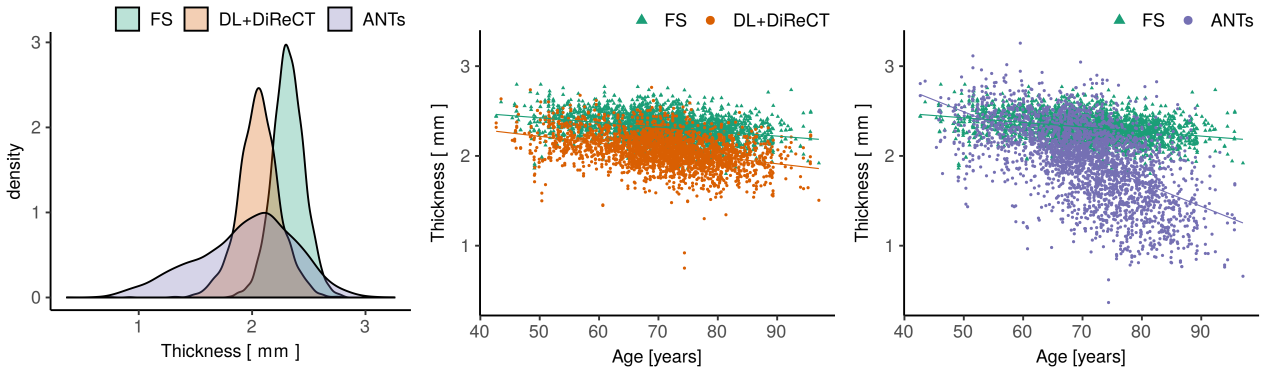

Pars triangularis - rh

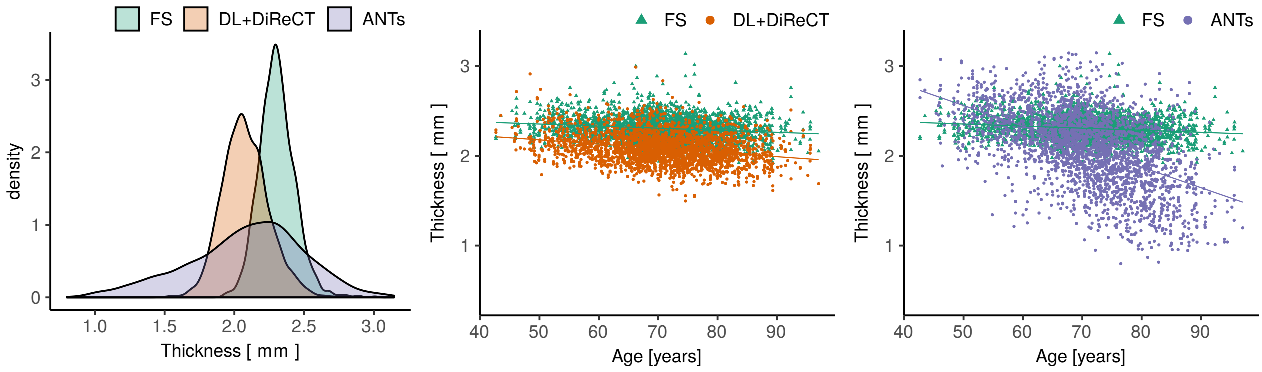

Pericalcerine cortex - lh

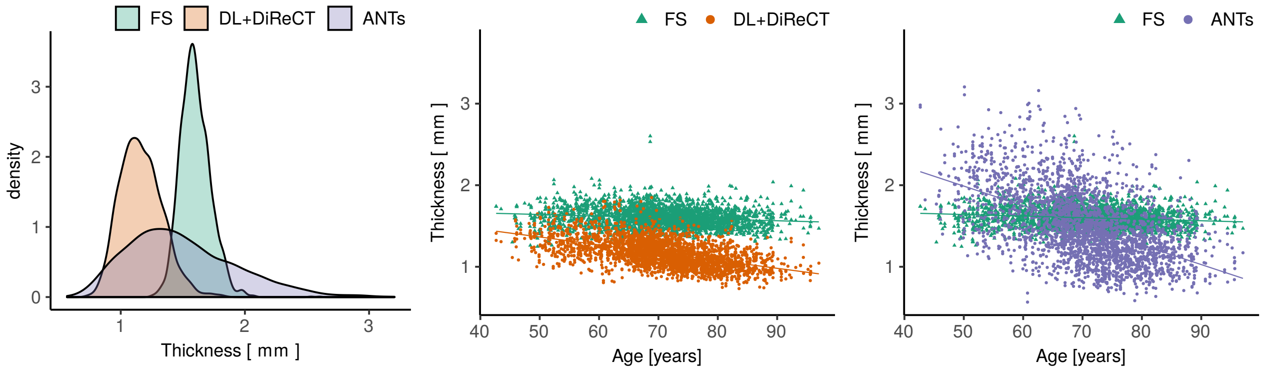

Pericalcerine cortex - rh

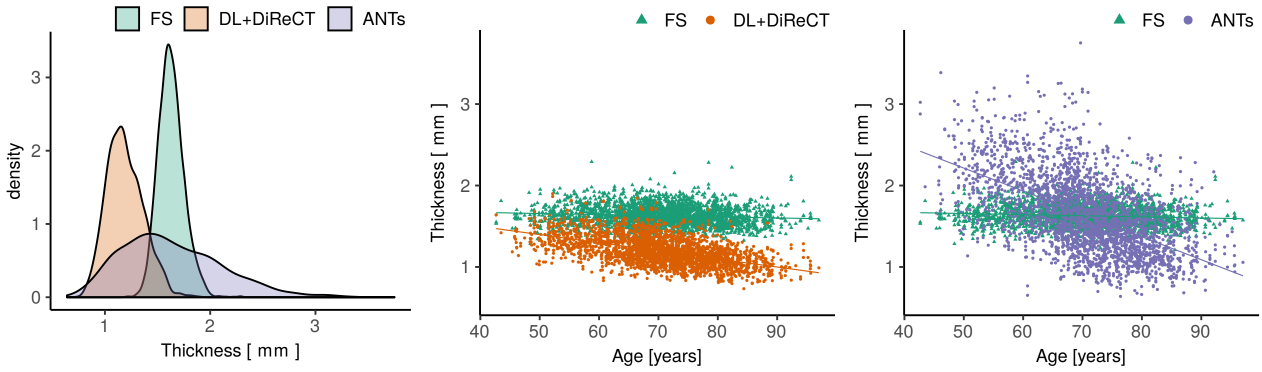

Postcentral gyrus - lh

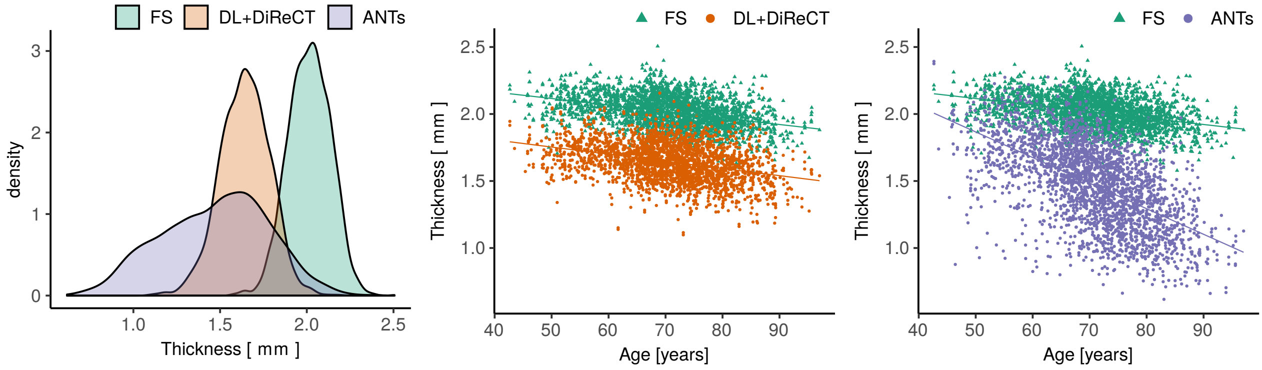

Postcentral gyrus - rh

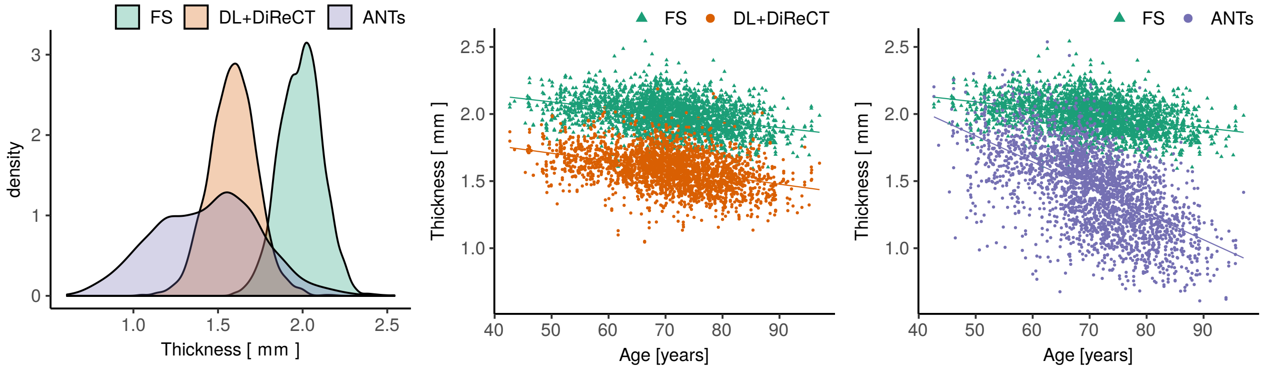

Posterior cingulate cortex - lh

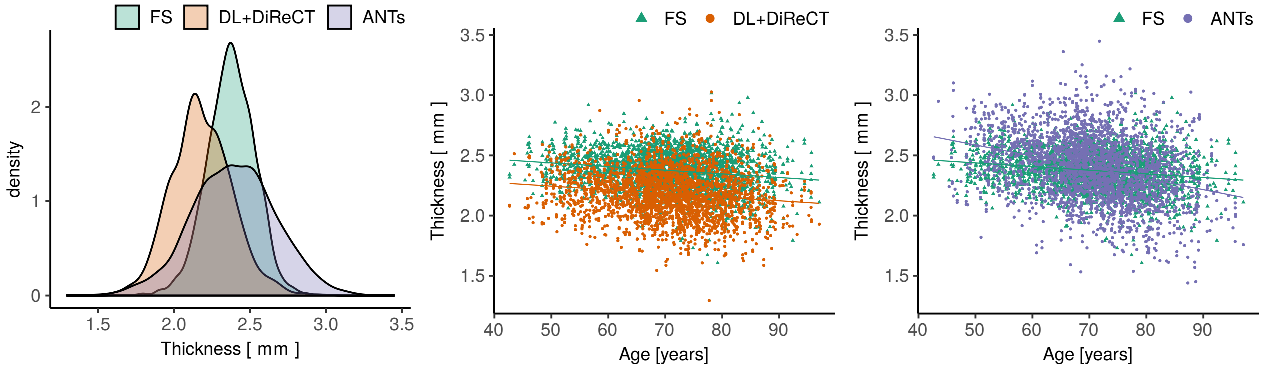

Posterior cingulate cortex - rh

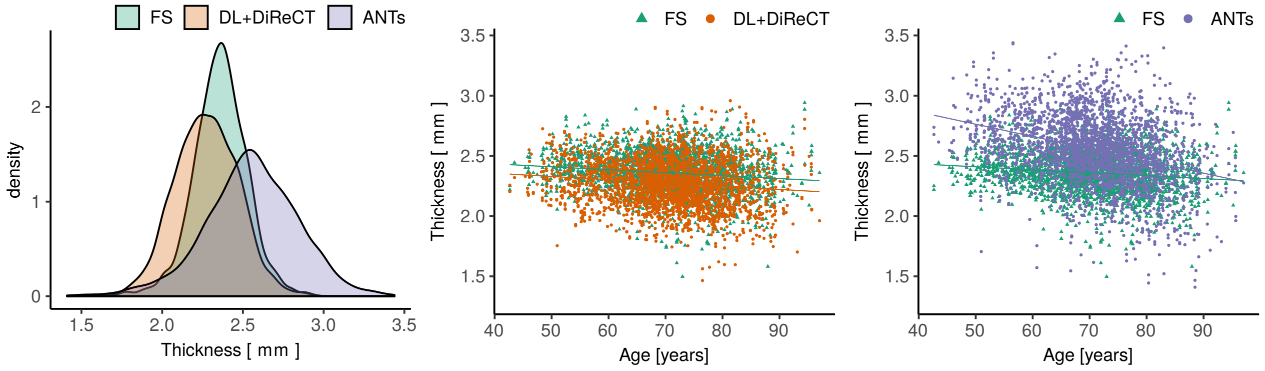

Precentral gyrus - lh

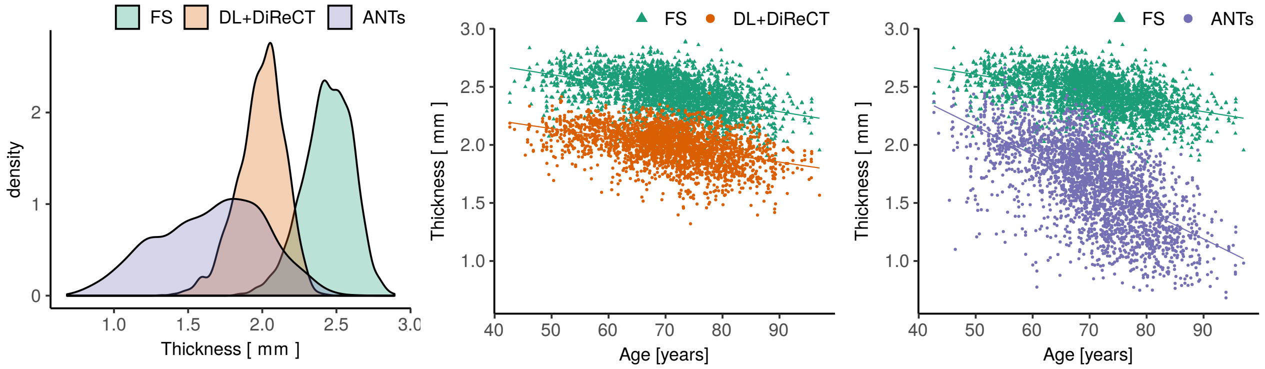

Precentral gyrus - rh

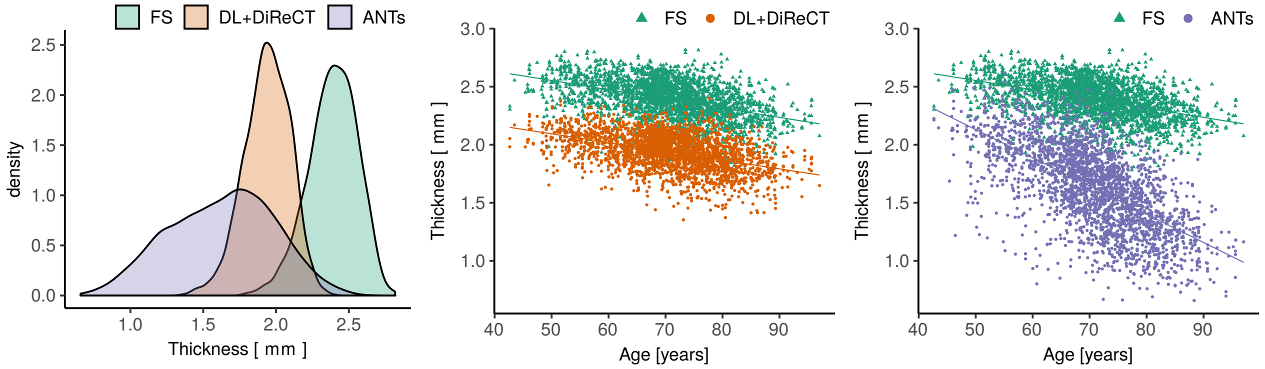

Precuneus - lh

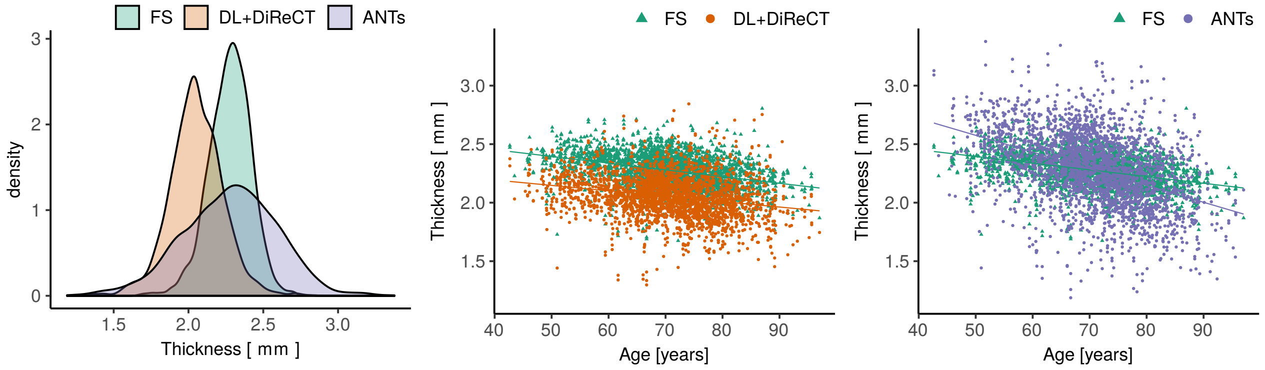

Precuneus - rh

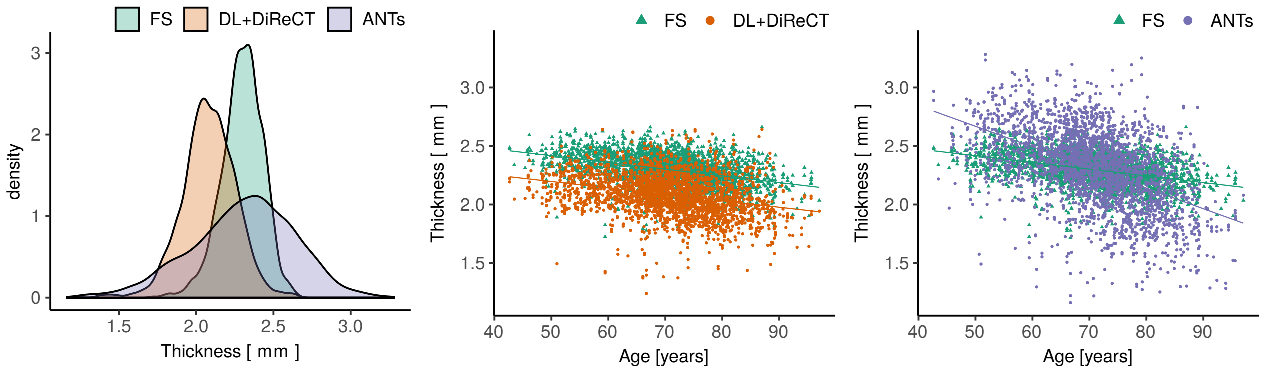

Rostral anterior cingulate cortex - lh

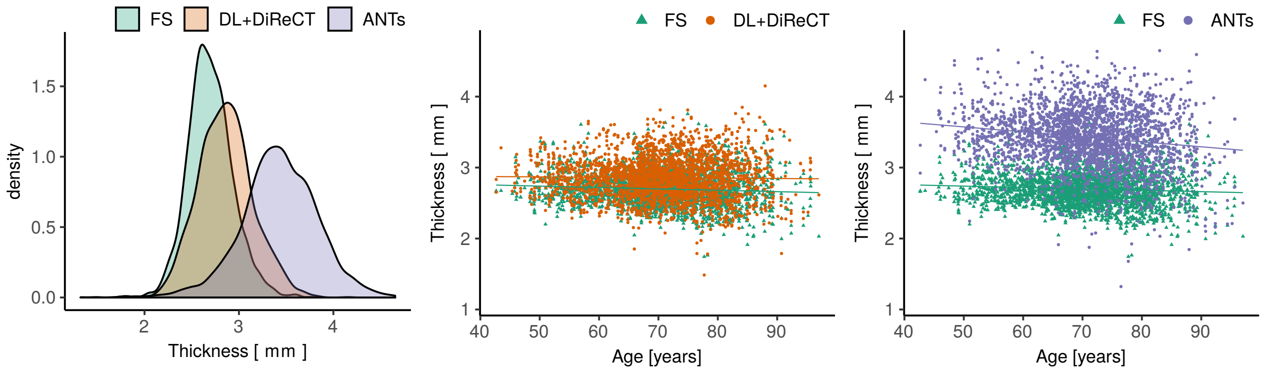

Rostral anterior cingulate cortex - rh

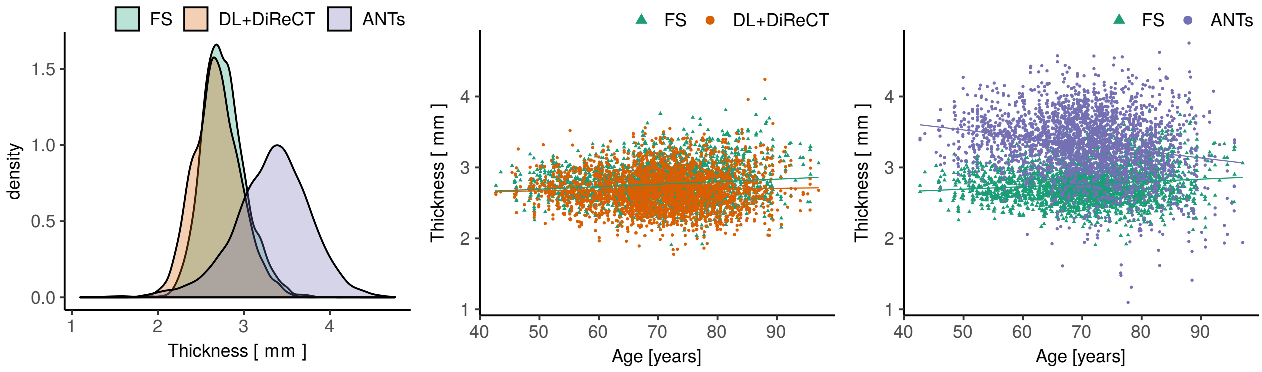

Rostral middle frontal cortex - lh

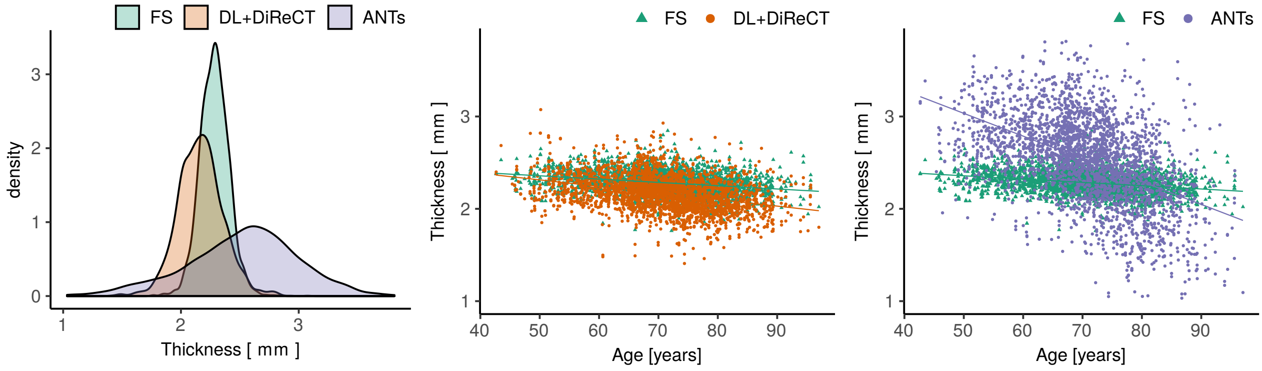

Rostral middle frontal cortex - rh

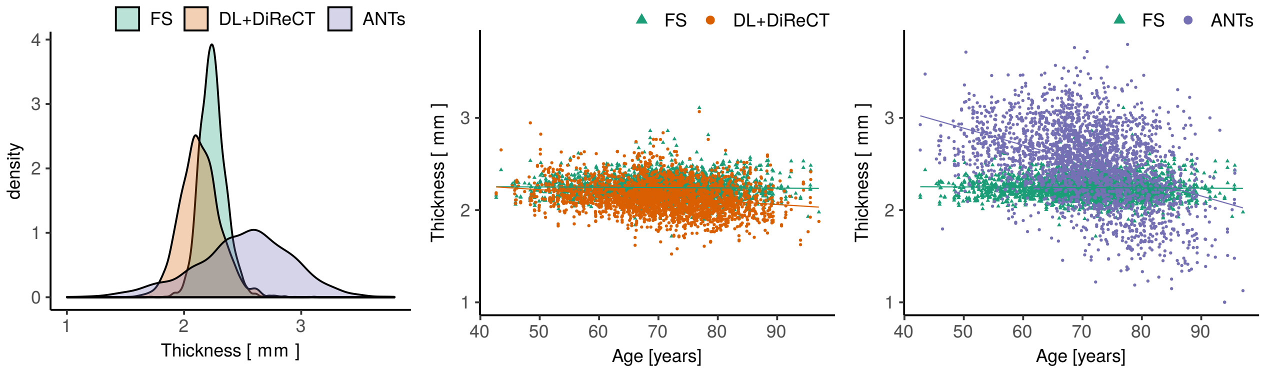

Superior frontal gyrus - lh

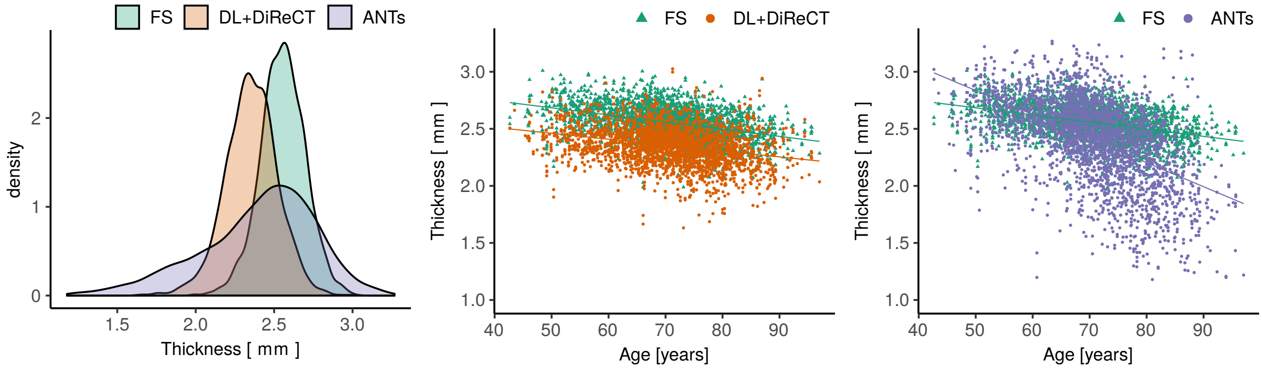

Superior frontal gyrus - rh

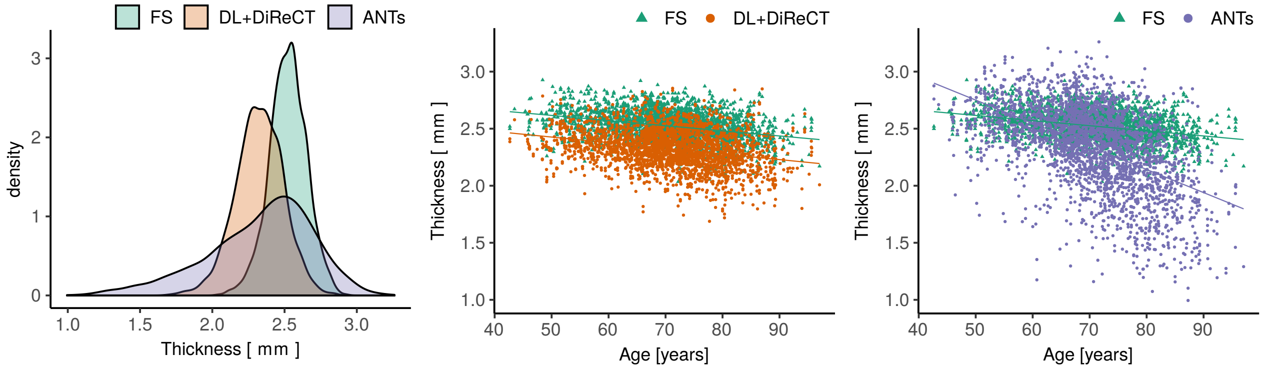

Superior parietal lobule - lh

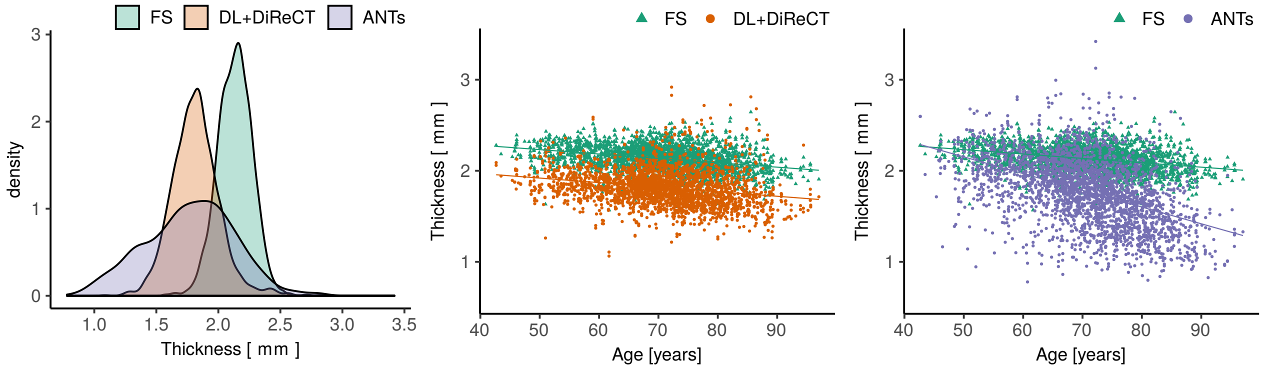

Superior parietal lobule - rh

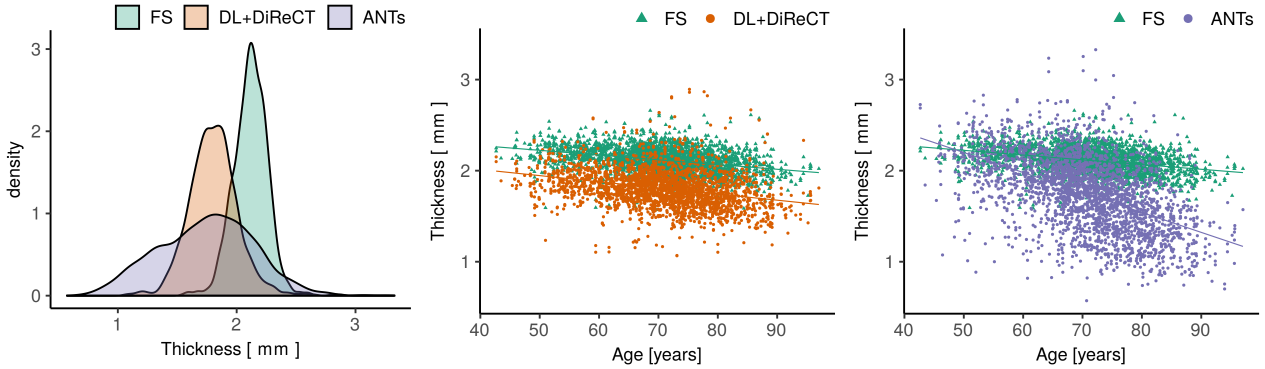

Superior temporal gyrus - lh

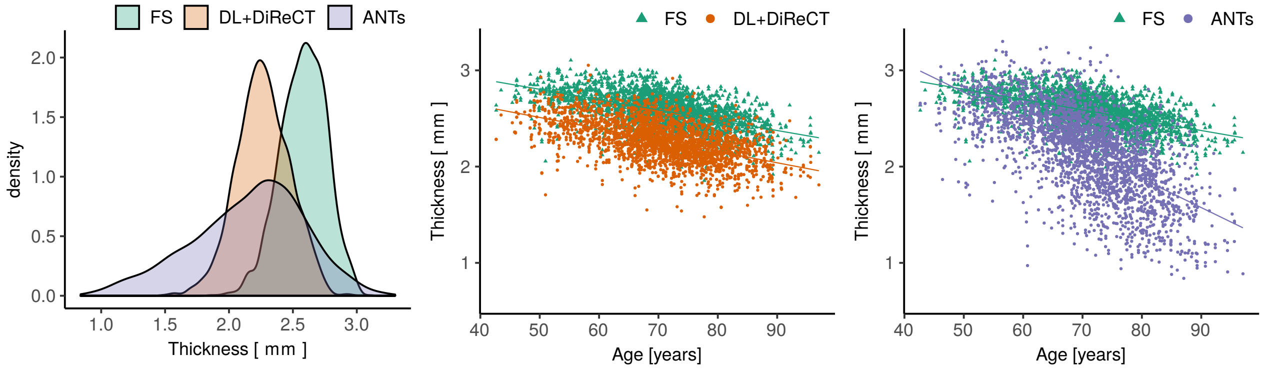

Superior temporal gyrus - rh

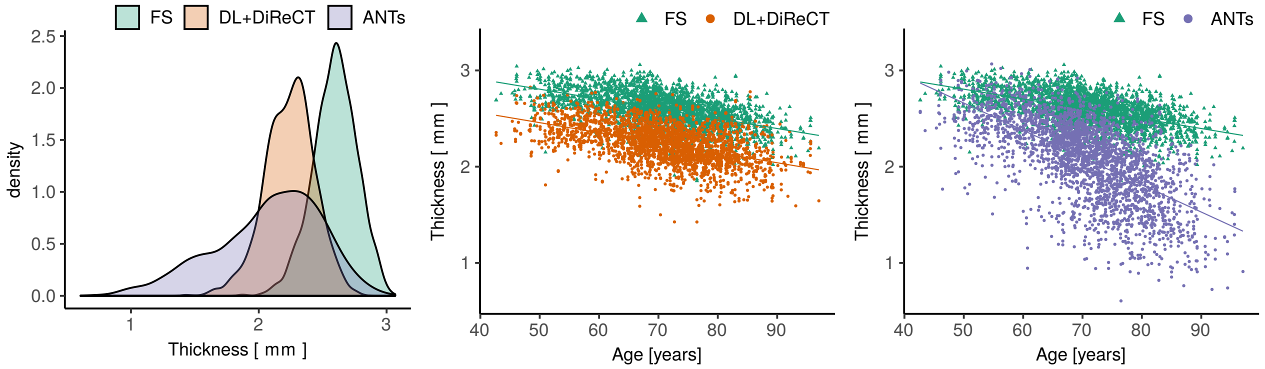

Supramarginal gyrus - lh

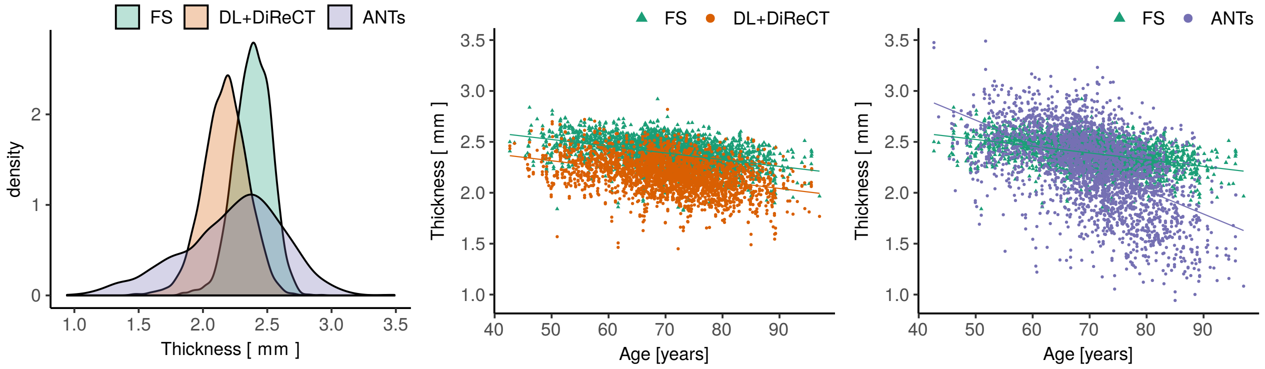

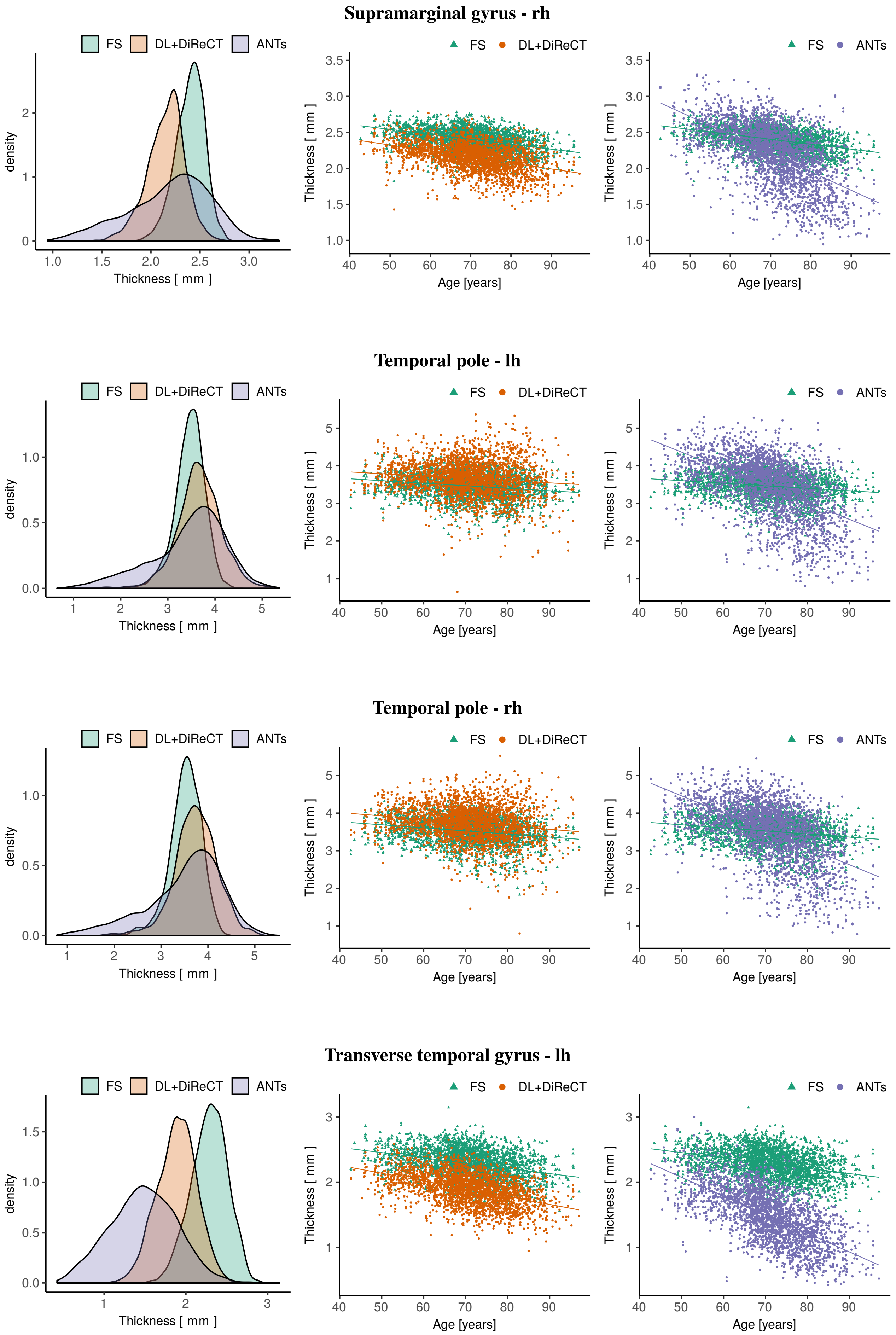
Supramarginal gyrus - rh
Temporal pole - lh
Temporal pole - rh
Transverse temporal gyrus - lh
FS
DL+DiReCT
ANTs
density
Thickness [mm]
Age [years]

Transverse temporal gyrus - rh

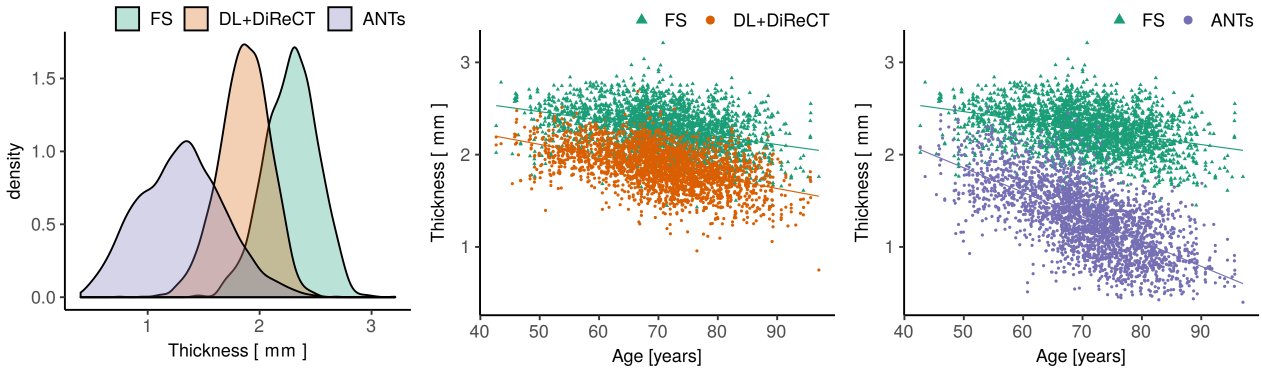

Global mean thickness - lh

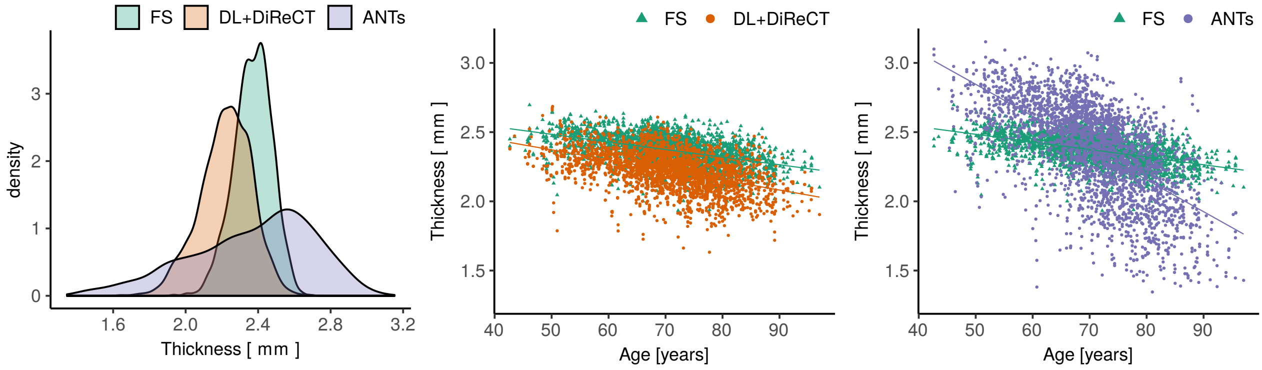

Global mean thickness - rh

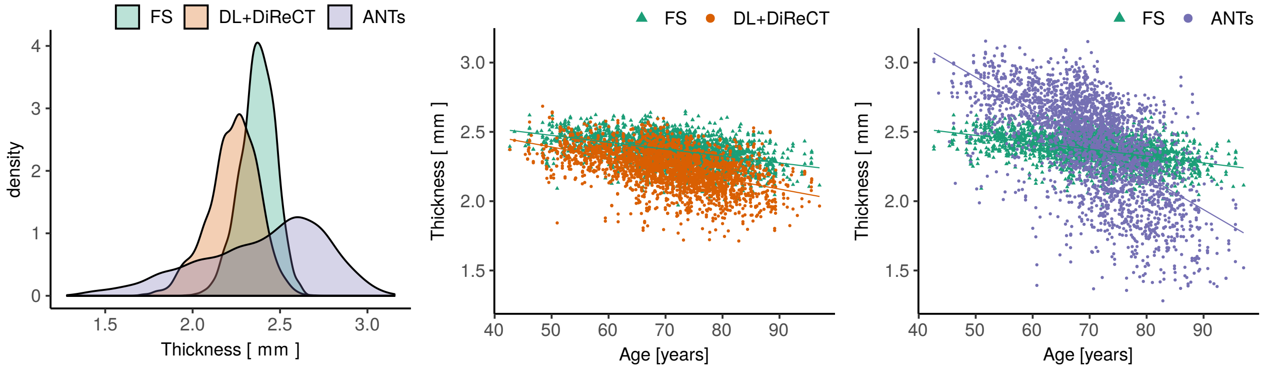

Supplement: Supplementary file 1 — Appendix S1: Supplementary Material [file HBM-41-4804-s001.pdf]
